# Supplementary material for: Active immunotherapy reduces NOTCH3 deposition in brain capillaries in a CADASIL mouse model
Source: EMBO Mol Med. 2022 Dec 16;15(2):e16556. doi: 10.15252/emmm.202216556 (PMC9906330; doi:10.15252/emmm.202216556)
Supplement: Supplementary file 1 — Appendix [file EMMM-15-e16556-s003.pdf]

Figure 4B		
**NOTCH3ECD deposits (% of vessel area)**		
**Comparison**	**Significance**	**Adjusted P Value**
7 m.o. vs. Sham	ns	0,9424
7 m.o. vs. Vaccinated	ns	0,9809
7 m.o. vs. 18 m.o.	**	0,0046
Sham vs. Vaccinated	ns	0,9999
Sham vs. 18 m.o.	**	0,0032
Vaccinated vs. 18 m.o.	**	0,0032
**NOTCH3ECD deposits (number/1000 um2)**		
**Comparison**	**Significance**	**Adjusted P Value**
7 m.o. vs. Sham	ns	0,7877
7 m.o. vs. Vaccinated	ns	0,8358
7 m.o. vs. 18 m.o.	**	0,0069
Sham vs. Vaccinated	ns	0,9995
Sham vs. 18 m.o.	**	0,0044
Vaccinated vs. 18 m.o.	*	0,0104
**NOTCH3ECD deposits size**		
**Comparison**	**Significance**	**Adjusted P Value**
7 m.o. vs. Sham	ns	0,1169
7 m.o. vs. Vaccinated	ns	>0.9999
7 m.o. vs. 18 m.o.	***	0,0009
Sham vs. Vaccinated	ns	0,0577
Sham vs. 18 m.o.	**	0,007
Vaccinated vs. 18 m.o.	****	<0.0001
**Figure 5B**		
**NOTCH3ECD deposits (% of vessel area)**		
**Comparison**	**Significance**	**Adjusted P Value**
3 m.o. vs. 18 m.o.	**	0,0029
3 m.o. vs. 7 m.o.	***	0,0004
7 m.o. vs. Sham	ns	0,7326
7 m.o. vs. Vaccinated	**	0,003
7 m.o. vs. 18 m.o.	****	<0.0001
Sham vs. Vaccinated	****	<0.0001

Sham vs. 18 m.o.	**	0,0014
Vaccinated vs. 18 m.o.	**	0,0046
**NOTCH3ECD deposits (number/1000 um2)**		
**Comparison**	**Significance**	**Adjusted P Value**
3 m.o. vs. 18 m.o.	**	0,0038
3 m.o. vs. 7 m.o.	***	0,0002
7 m.o. vs. Sham	ns	0,9913
7 m.o. vs. Vaccinated	*	0,021
7 m.o. vs. 18 m.o.	**	0,0043
Sham vs. Vaccinated	***	0,001
Sham vs. 18 m.o.	**	0,0041
Vaccinated vs. 18 m.o.	*	0,0117
**NOTCH3ECD deposits size**		
**Comparison**	**Significance**	**Adjusted P Value**
3 m.o. vs. 18 m.o.	*	0,0215
3 m.o. vs. 7 m.o.	**	0,0029
7 m.o. vs. Sham	ns	0,3622
7 m.o. vs. Vaccinated	*	0,0428
7 m.o. vs. 18 m.o.	*	0,0196
Sham vs. Vaccinated	**	0,0038
Sham vs. 18 m.o.	*	0,0469
Vaccinated vs. 18 m.o.	*	0,0329
**Figure 6A**		
**Serum hN3ECD protein**		
**Comparison**	**Significance**	**Adjusted P Value**
3 m.o. vs 7 m.o.	***	0,0005
**Figure 6B**		
**Serum hN3ECD protein**		
**Comparison**	**Significance**	**Adjusted P Value**
Sham vs Vaccinated	*	0,0196
**Figure 7B**		
**% microglia with CD68 Staining**		
**Comparison**	**Significance**	**Adjusted P Value**

7 m.o. vs. Sham	ns	0,7393
7 m.o. vs. Vaccinated	*	0,0251
Sham vs. Vaccinated	**	0,0087
**CD68 Staining (% of microglia area)**		
**Comparison**	**Significance**	**Adjusted P Value**
7 m.o. vs. Sham	ns	0,9131
7 m.o. vs. Vaccinated	*	0,0239
Sham vs. Vaccinated	*	0,0157
**Figure 7D**		
**% microglia with NOTCH3ECD deposits**		
**Comparison**	**Significance**	**Adjusted P Value**
7 m.o. vs. Sham	ns	0,7997
7 m.o. vs. Vaccinated	ns	0,2685
Sham vs. Vaccinated	ns	0,0526
**NOTCH3ECD deposits (% of microglia area)**		
**Comparison**	**Significance**	**Adjusted P Value**
7 m.o. vs. Sham	ns	0,5362
7 m.o. vs. Vaccinated	ns	0,2186
Sham vs. Vaccinated	ns	0,8059
**NOTCH3ECD deposits (number/1000 um2)**		
**Comparison**	**Significance**	**Adjusted P Value**
7 m.o. vs. Sham	ns	0,8392
7 m.o. vs. Vaccinated	ns	0,2974
Sham vs. Vaccinated	ns	0,5777
**NOTCH3ECD deposits size**		
**Comparison**	**Significance**	**Adjusted P Value**
7 m.o. vs. Sham	ns	0,6664
7 m.o. vs. Vaccinated	ns	0,3748
Sham vs. Vaccinated	ns	0,8787
**Figure EV3**		
**Serum hN3ECD protein**		
**Comparison**	**Significance**	**Adjusted P Value**
TgN3R182C150 vs C57BL6/J WT Sham	ns	0,8383
TgN3R182C150 vs C57BL6/J WT Vaccinated	ns	0,7

Figure EV4C		
**Serum CRP concentration**		
**Comparison**	**Significance**	**Adjusted P Value**
Vaccinated vs. Sham	ns	0,7002
**Figure EV4D**		
**Serum CRP concentration**		
**Comparison**	**Significance**	**Adjusted P Value**
Sham vs. Vaccinated	ns	0,9974
Sham vs. 7 m.o.	ns	0,518
Vaccinated vs. 7 m.o.	ns	0,5638
**Figure EV5**		
**RLU (relative luceferin units)**		
**Comparison**	**Significance**	**Adjusted P Value**
**Control**		
Fc +DMSO vs. Fc + DAPT	ns	>0.9999
Fc +DMSO vs. Jag2 + DMSO	ns	0,7223
Fc +DMSO vs. Jag2 + DAPT	ns	0,9982
Fc + DAPT vs. Jag2 + DMSO	ns	0,7225
Fc + DAPT vs. Jag2 + DAPT	ns	0,9982
Jag2 + DMSO vs. Jag2 + DAPT	ns	0,817
**N3WT**		
Fc +DMSO vs. Fc + DAPT	ns	0,9613
Fc +DMSO vs. Jag2 + DMSO	***	0,0004
Fc +DMSO vs. Jag2 + DAPT	ns	0,8237
Fc + DAPT vs. Jag2 + DMSO	****	<0.0001
Fc + DAPT vs. Jag2 + DAPT	ns	0,5338
Jag2 + DMSO vs. Jag2 + DAPT	**	0,0069
**N3R182C**		
Fc +DMSO vs. Fc + DAPT	ns	0,9993
Fc +DMSO vs. Jag2 + DMSO	*	0,0135
Fc +DMSO vs. Jag2 + DAPT	ns	0,9841
Fc + DAPT vs. Jag2 + DMSO	**	0,0094
Fc + DAPT vs. Jag2 + DAPT	ns	0,9621
Jag2 + DMSO vs. Jag2 + DAPT	*	0,0352

**Jag2 + DMSO**		
Control vs. N3WT	***	0,0008
Control vs. N3R182C	*	0,0314
N3WT vs. N3R182C	ns	0,4187
