# Supplementary material for: Active immunotherapy reduces NOTCH3 deposition in brain capillaries in a CADASIL mouse model
Source: EMBO Mol Med. 2022 Dec 16;15(2):e16556. doi: 10.15252/emmm.202216556 (PMC9906330; doi:10.15252/emmm.202216556)
Supplement: Supplementary file 2 — Expanded View Figures PDF [file EMMM-15-e16556-s002.pdf]

Expanded View Figures

Figure EV1. Smooth muscle Actin (ASMA) is expressed in arteries/arterioles but not in capillaries.

Immunofluorescence analysis of the expression of ASMA, Perlecan, PDGFRβ and NG2 in isolated mouse brain vessels from TgN3R182C[150] and WT mouse as indicated. Nuclei are stained with DAPI. Note the more restricted staining of SMA in arteries/arterioles but not in pericytes, while PDGFRβ, NG2 and perlecan staining is more extensive (PDGFRβ and NG2 stain both VSMC and pericytes and perlecan stain VSMC, pericytes and endothelial cells). Scale bar = 40 μm.

Source data are available online for this figure.

© 2022 The Authors

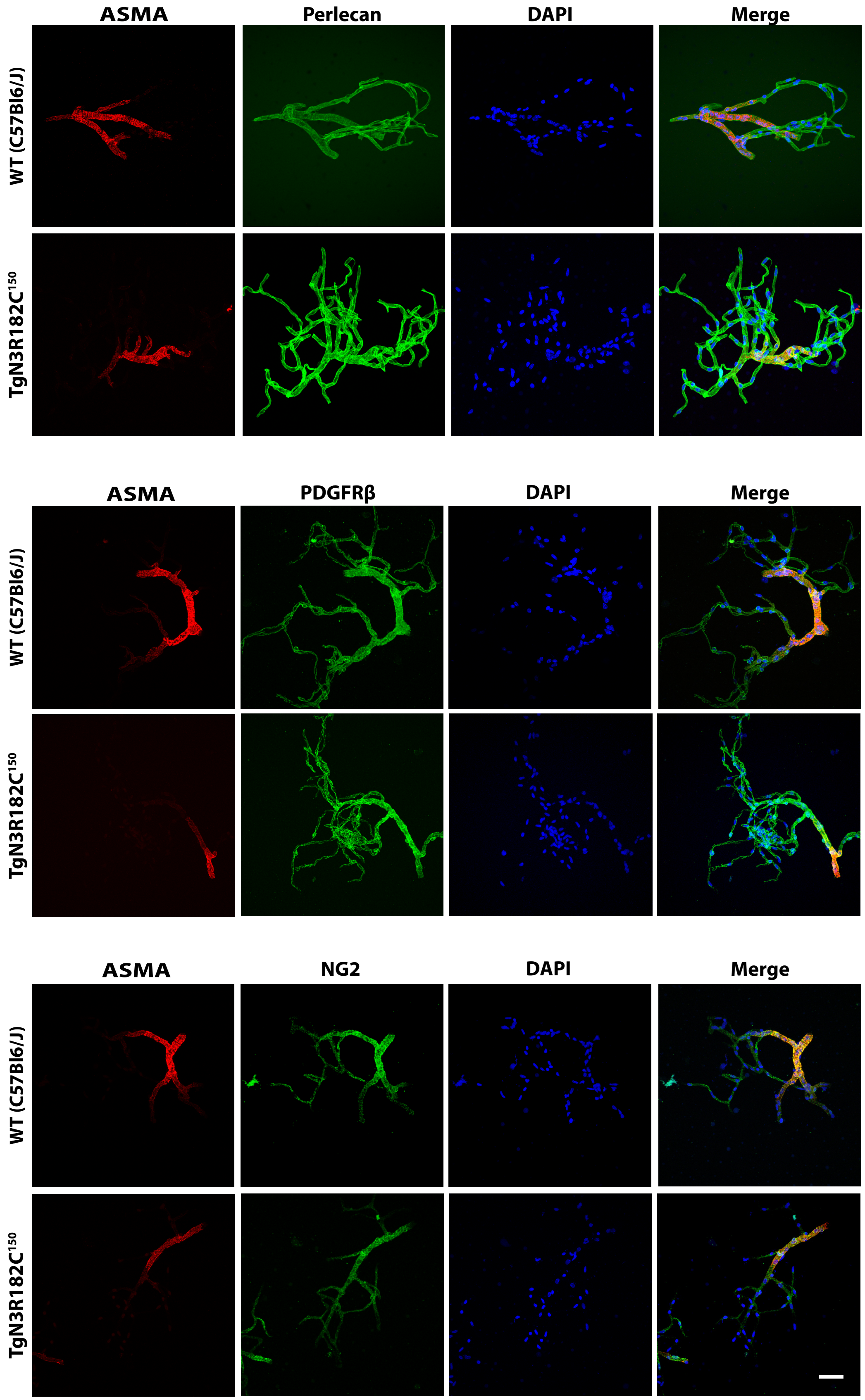

Figure EV1.

 © 2022 The Authors

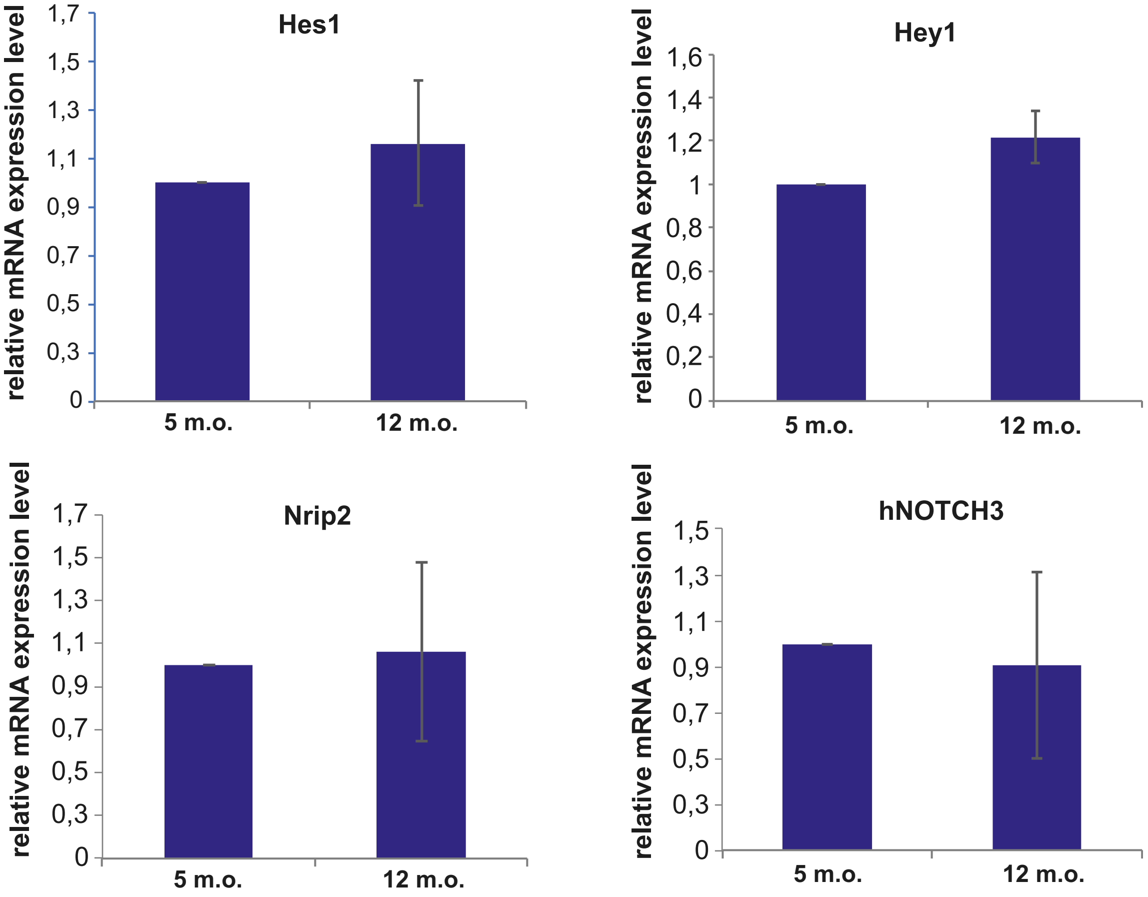

Figure EV2. Quantitative real-time PCR analysis of the Notch downstream target genes.

The target genes *NOTCH3*, *Hes1*, *Hey1* and *Nrip2* on TgN3R182C150 mice at 5 and 12 months of age. Three biological replicates were run in three technical replicates. Error bars indicate standard deviation (SD).

© 2022 The Authors

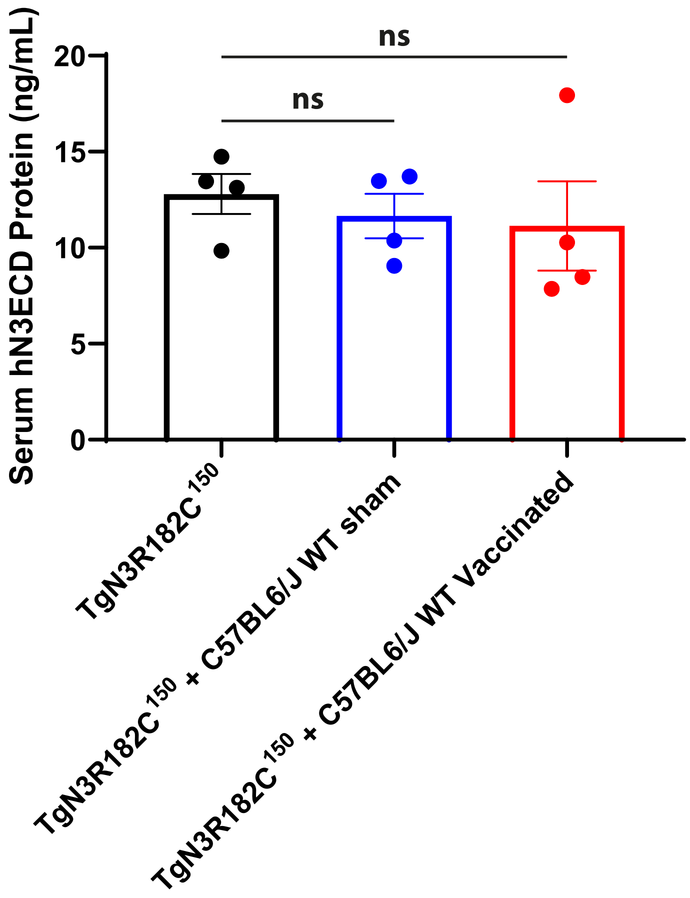

Figure EV3. NOTCH3 ECD protein competitive ELISA assay.

Serum from 7-month-old non-vaccinated TgN3-R182C^{150} mice (n = 4) was monitored in the absence or presence of serum (1:1 dilution) from sham-and vaccinated C57Bl6/J WT mice. No significant changes in NOTCH3 ECD concentration was observed after dilution adjustment. Statistical significance was assessed using an ordinary one-way ANOVA followed by Dunnett's multiple comparisons test (TgN3R182C^{150} vs. TgN3R182C^{150} + C57BL6/J WT Sham, ns P = 0.8383; TgN3R182C^{150} vs. TgN3R182C^{150} + C57BL6/J WT Vaccinated, ns P = 0.7000; ns = non-significant). Error bars indicate standard error of the mean (SEM).

Source data are available online for this figure.

Figure EV4. Safety and toxicity evaluation of the active immunotherapy.

A At the top, representative images of H&E staining of two vaccinated and two sham mice show normal-looking structure of kidneys from sham and vaccinated mice, as indicated. Arrows show glomeruli and arrowheads show tubuli. At the bottom, cleaved-Caspase3 staining on kidney sections from sham and vaccinated mice is shown, with no sign of damage (each biological replicate was run in five technical replicates). Apoptotic cells are indicated by arrows. Scale bar = 50 µm.

B Serum CRP concentrations were performed in duplicate in sham (n = 9) and vaccinated (n = 8) mice as determined by ELISA. Statistical significance was assessed by unpaired Student's t-test with welch's correction (Vaccinated vs. Sham ns P = 0.7002, ns = non-significant). Error bars indicate standard error of the mean (SEM).

C Serum Neurofilament Light chain concentrations in sham (n = 8), vaccinated (n = 8), and non-vaccinated (n = 12) mice was determined by SIMOA. Statistical significance was assessed using an ordinary one-way ANOVA followed by Tukey's multiple comparisons test (Sham vs. Vaccinated ns P = 0.9974; Sham vs. 7 m.o. ns P = 0.518; Vaccinated vs. 7 m.o. ns P = 0.5638, ns = non-significant). Error bars indicate standard error of the mean (SEM).

Source data are available online for this figure.

▶

 © 2022 The Authors

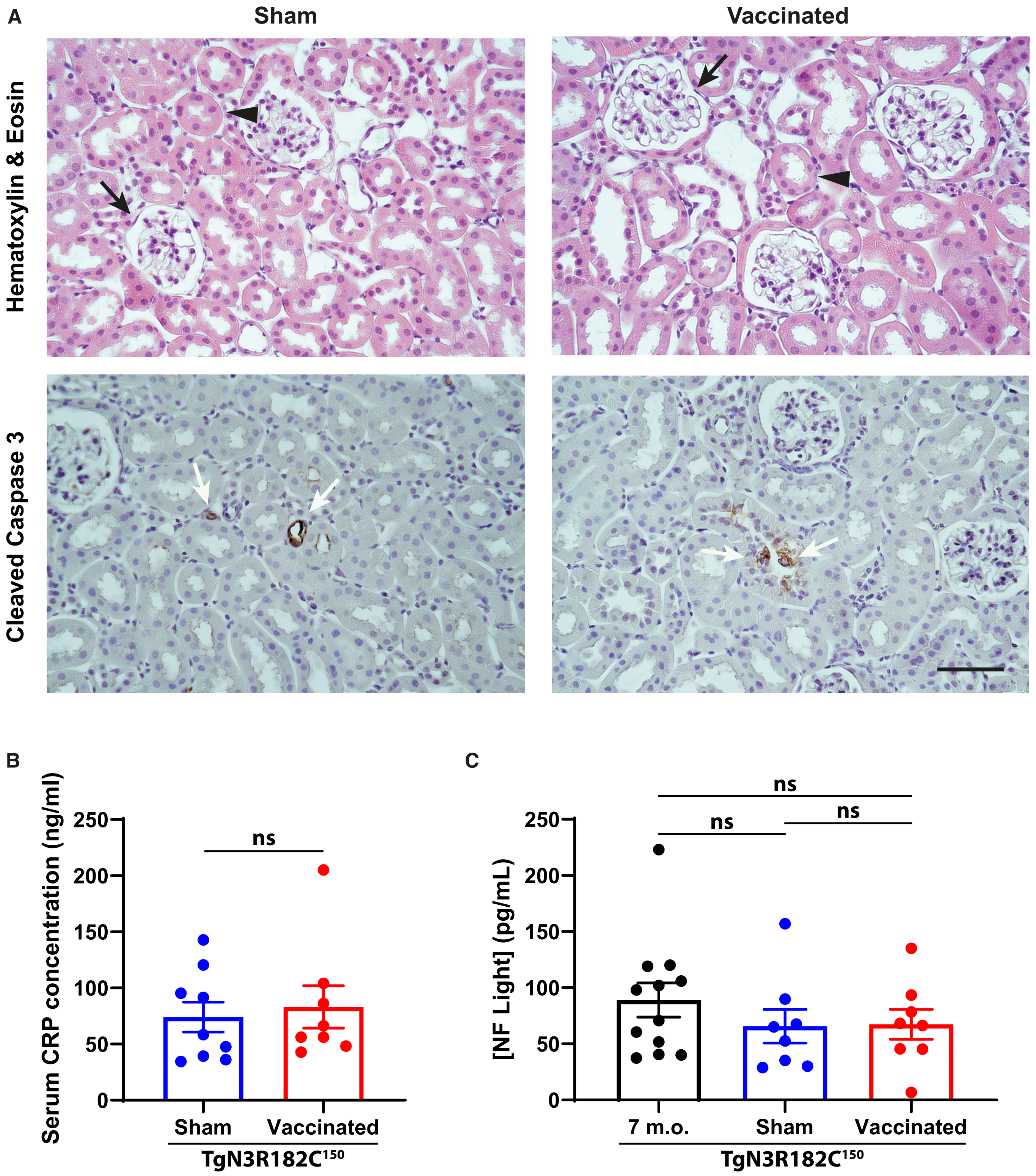
A
Sham
Vaccinated
Hematoxylin & Eosin
Cleaved Caspase 3
B
Serum CRP concentration (ng/ml)
250
200
150
100
50
0
ns
Sham
Vaccinated
TgN3R182C150
C
[NF Light] (pg/mL)
ns
ns
ns
7 m.o.
Sham
Vaccinated
TgN3R182C150

Figure EV4.

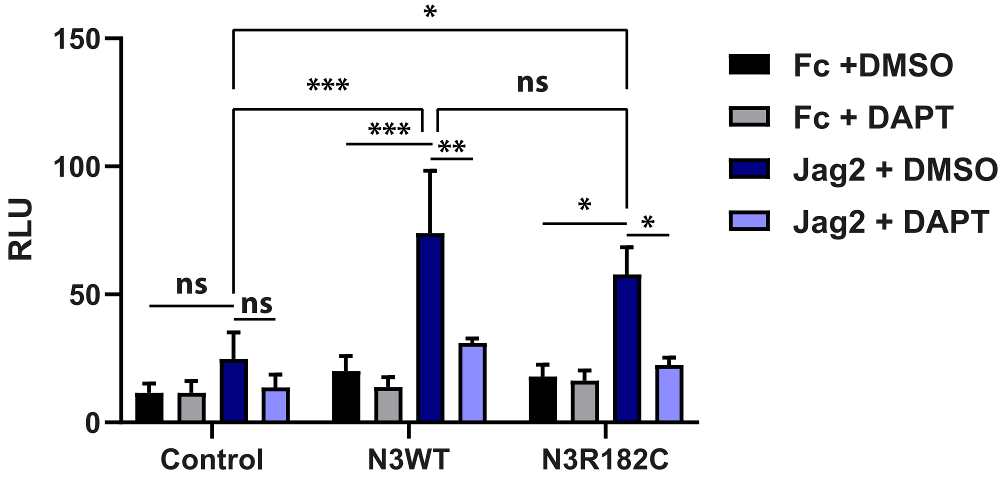

Figure EV5. NOTCH3 R182C mutation is signaling neutral.

NIH3T3 cells were transfected with the control, WT NOTCH3, or NOTCH3 R182C plasmids, as well as the β-gal and 12XCSL-luc reporter plasmids and cultured on immobilized jagged2 (Jag2) in the presence of DMSO or DAPT ($n = 3$ and two technical replicates). Statistical analysis was performed using 2-way ANOVA followed by Tukey's multiple comparisons tests (Control: Fc + DMSO vs. Jag2 + DMSO ns $P = 0.7223$; Jag2 + DMSO vs. Jag2 + DAPT ns $P = 0.817$. N3WT: Fc + DMSO vs. Jag2 + DMSO ***$P = 0.0004$; Jag2 + DMSO vs. Jag2 + DAPT **$P = 0.0069$. N3R182C: Fc + DMSO vs. Jag2 + DMSO *$P = 0.0135$; Jag2 + DMSO vs. Jag2 + DAPT *$P = 0.0352$. Jag2 + DMSO: Control Jag2 + DMSO vs. N3WT Jag2 + DMSO ***$P = 0.0008$; Control Jag2 + DMSO vs. N3R182C Jag2 + DMSO *$P = 0.0314$; N3WT Jag2 + DMSO vs. N3R182C Jag2 + DMSO ns $P = 0.4187$, ns = non-significant). Error bars indicate standard error of the mean (SEM). RLU, relative luminescence units.

Source data are available online for this figure.

 © 2022 The Authors
