# Supplementary figures and images for: Active immunotherapy reduces NOTCH3 deposition in brain capillaries in a CADASIL mouse model
Source: EMBO Mol Med. 2022 Dec 16;15(2):e16556. doi: 10.15252/emmm.202216556 (PMC9906330; doi:10.15252/emmm.202216556)

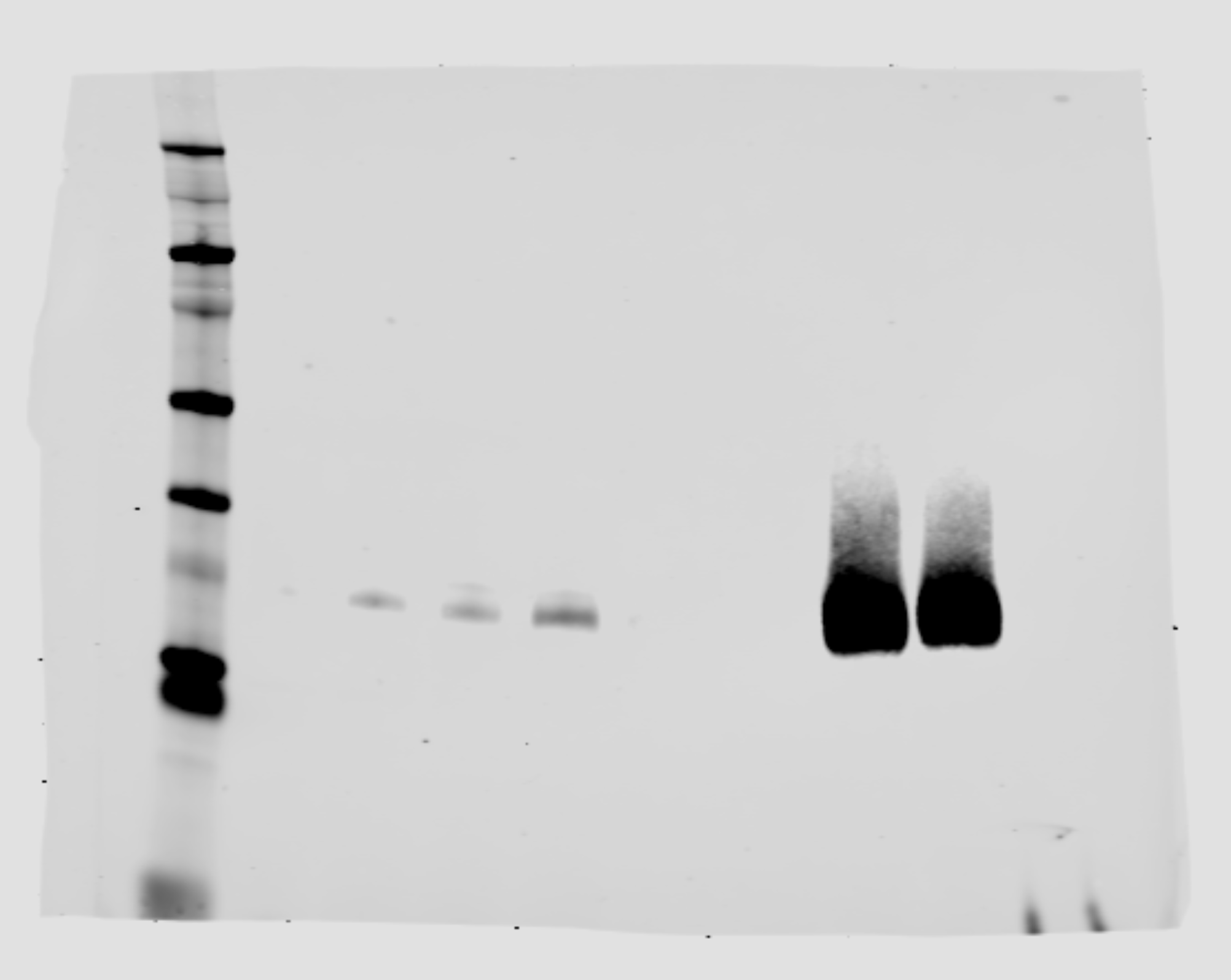

Supplement: Supplementary file 5 — Source Data for Figure 2 [file EMMM-15-e16556-s010.zip › EMMM_2423_Fig2B_full_membrane.tif]

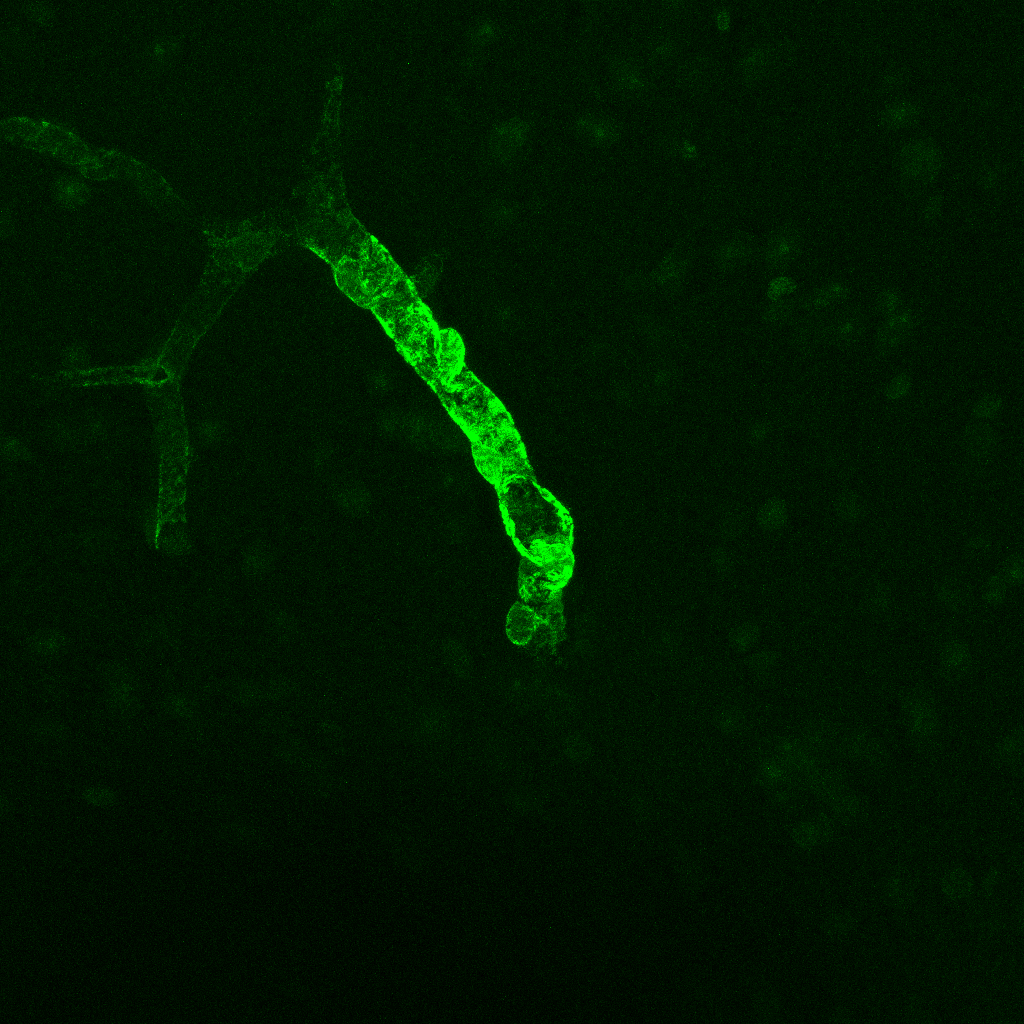

Supplement: Supplementary file 6 — Source Data for Figure 4 [file EMMM-15-e16556-s005.zip › Source data Fig4images 2/Sham_A6_image1_green_final.tif]

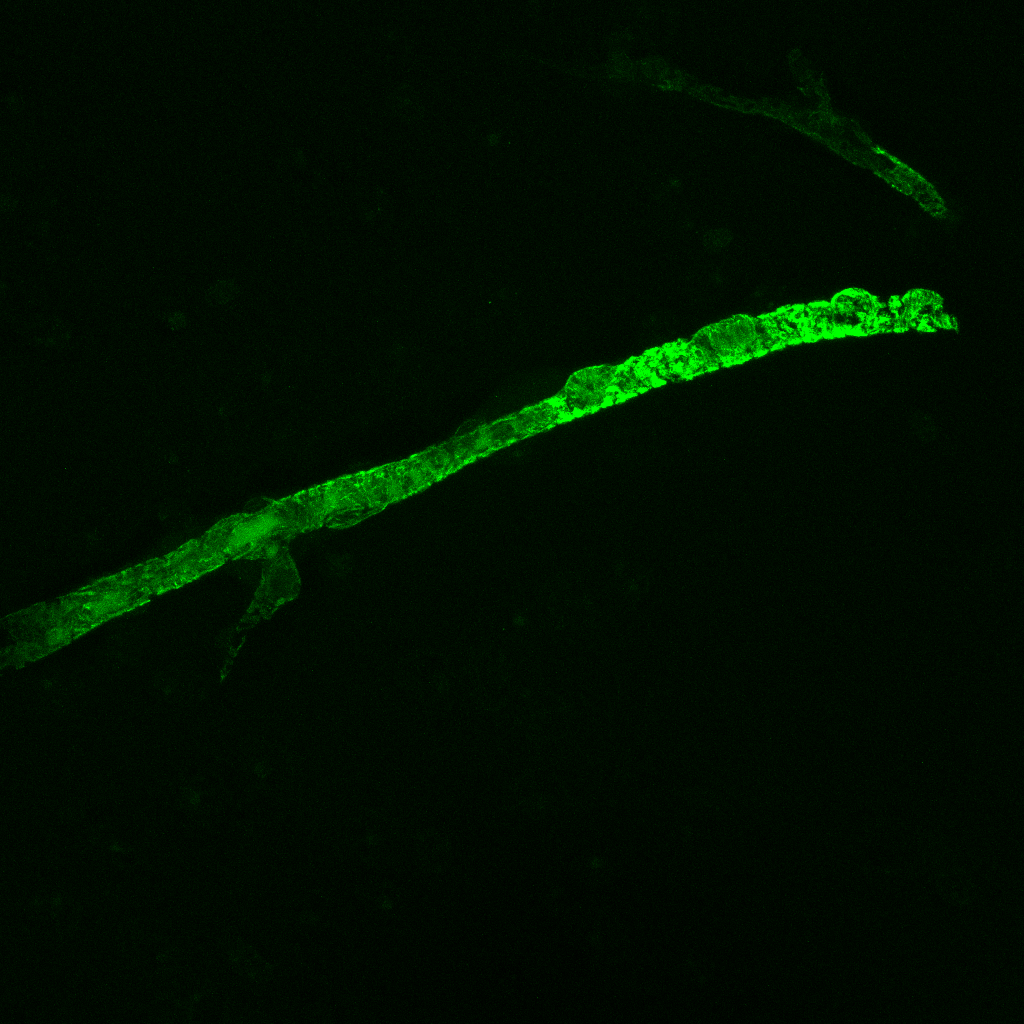

Supplement: Supplementary file 6 — Source Data for Figure 4 [file EMMM-15-e16556-s005.zip › Source data Fig4images 2/18mo_TG18_image5_greenv3_final.tif]

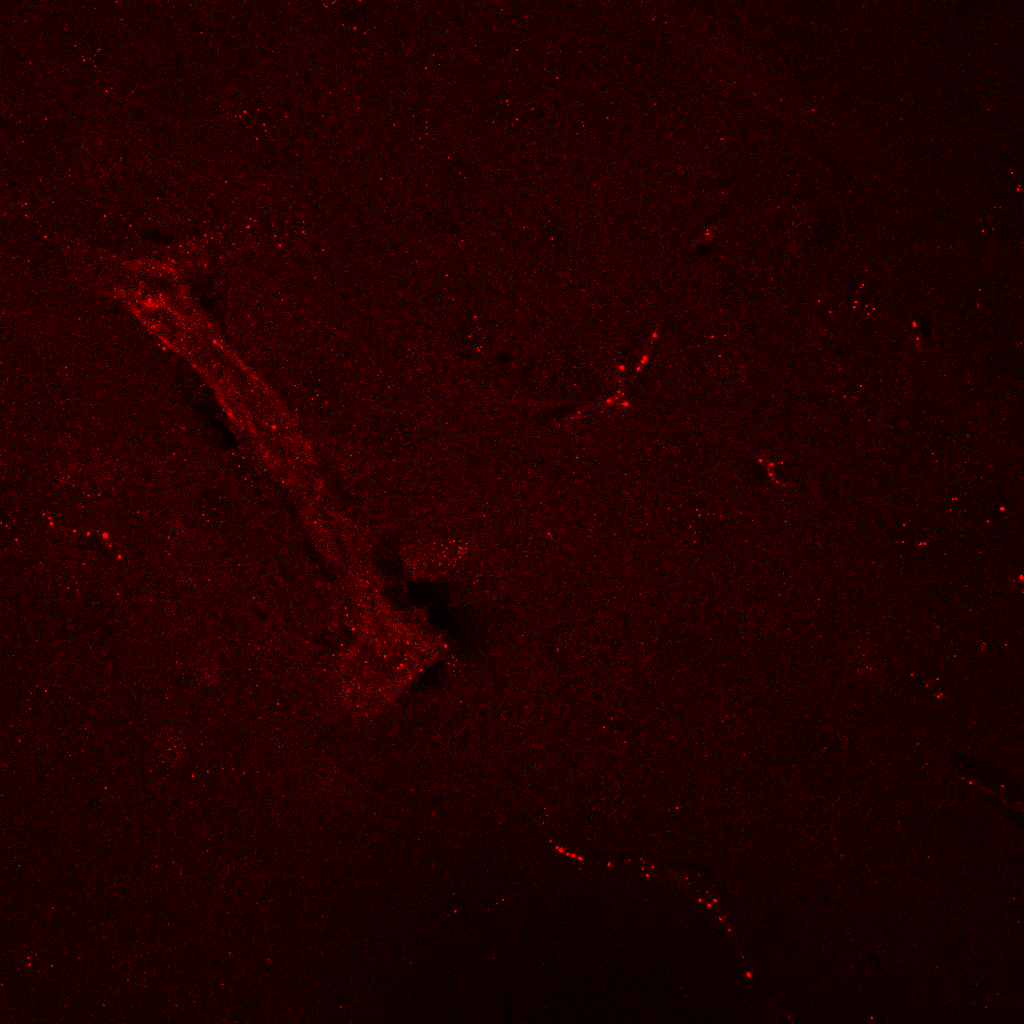

Supplement: Supplementary file 6 — Source Data for Figure 4 [file EMMM-15-e16556-s005.zip › Source data Fig4images 2/Sham_C2_image1_redv2.tif]

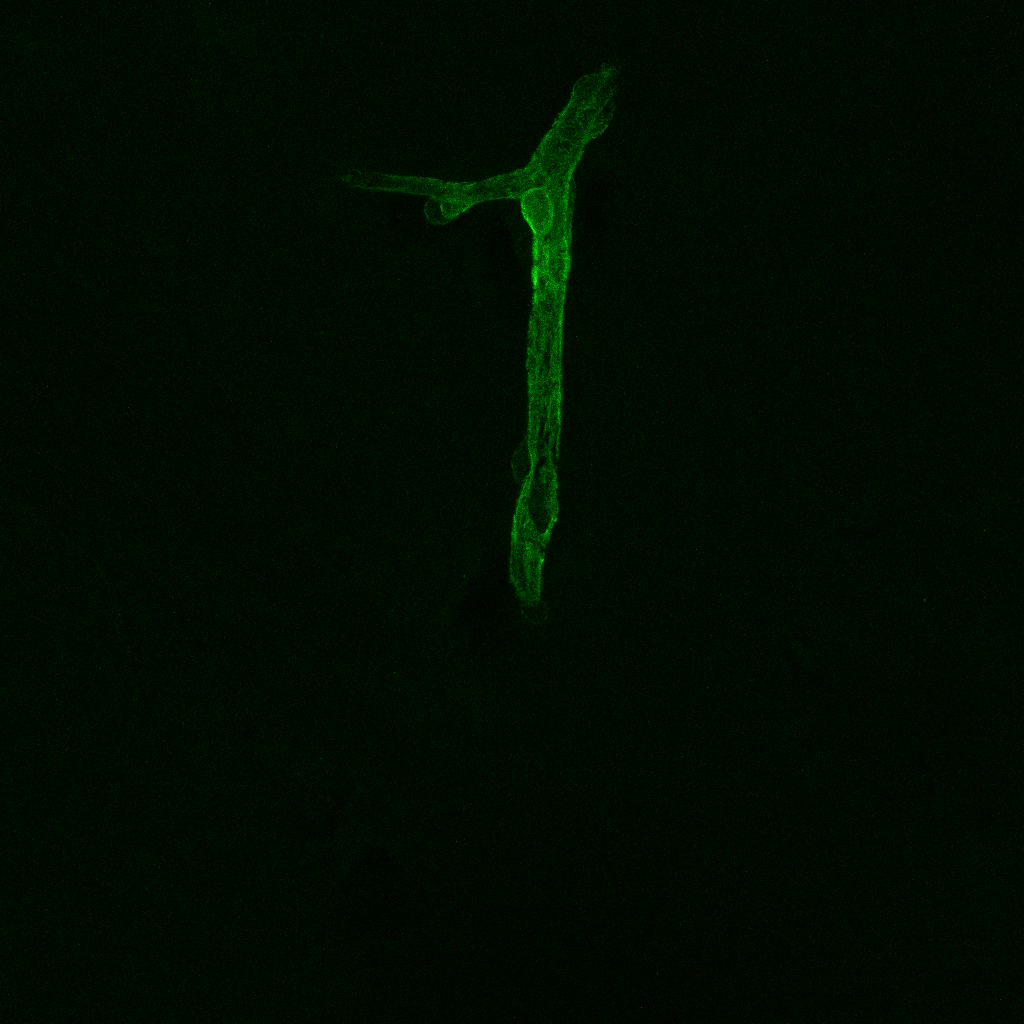

Supplement: Supplementary file 6 — Source Data for Figure 4 [file EMMM-15-e16556-s005.zip › Source data Fig4images 2/7mo_Image 1_asma_TG1_greenfinal.tif]

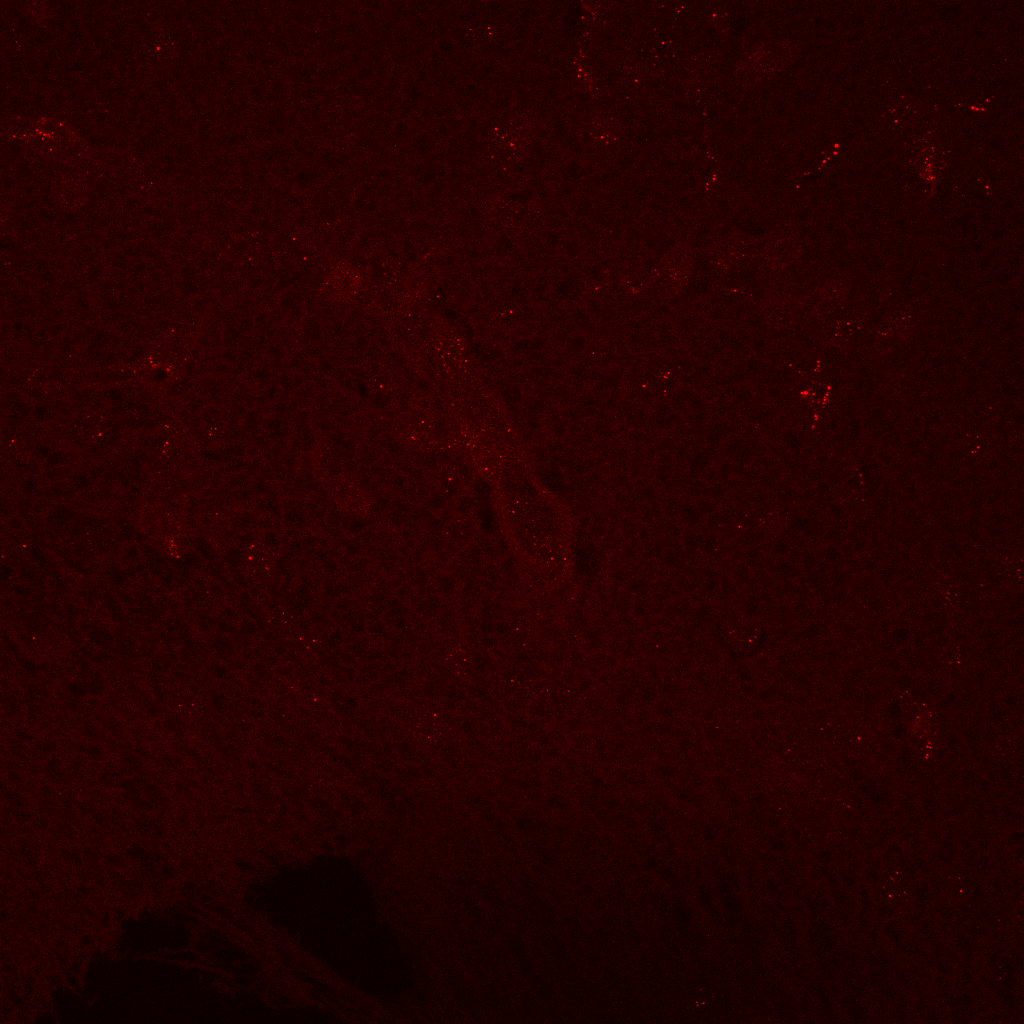

Supplement: Supplementary file 6 — Source Data for Figure 4 [file EMMM-15-e16556-s005.zip › Source data Fig4images 2/Sham_A6_image1_red.tif]

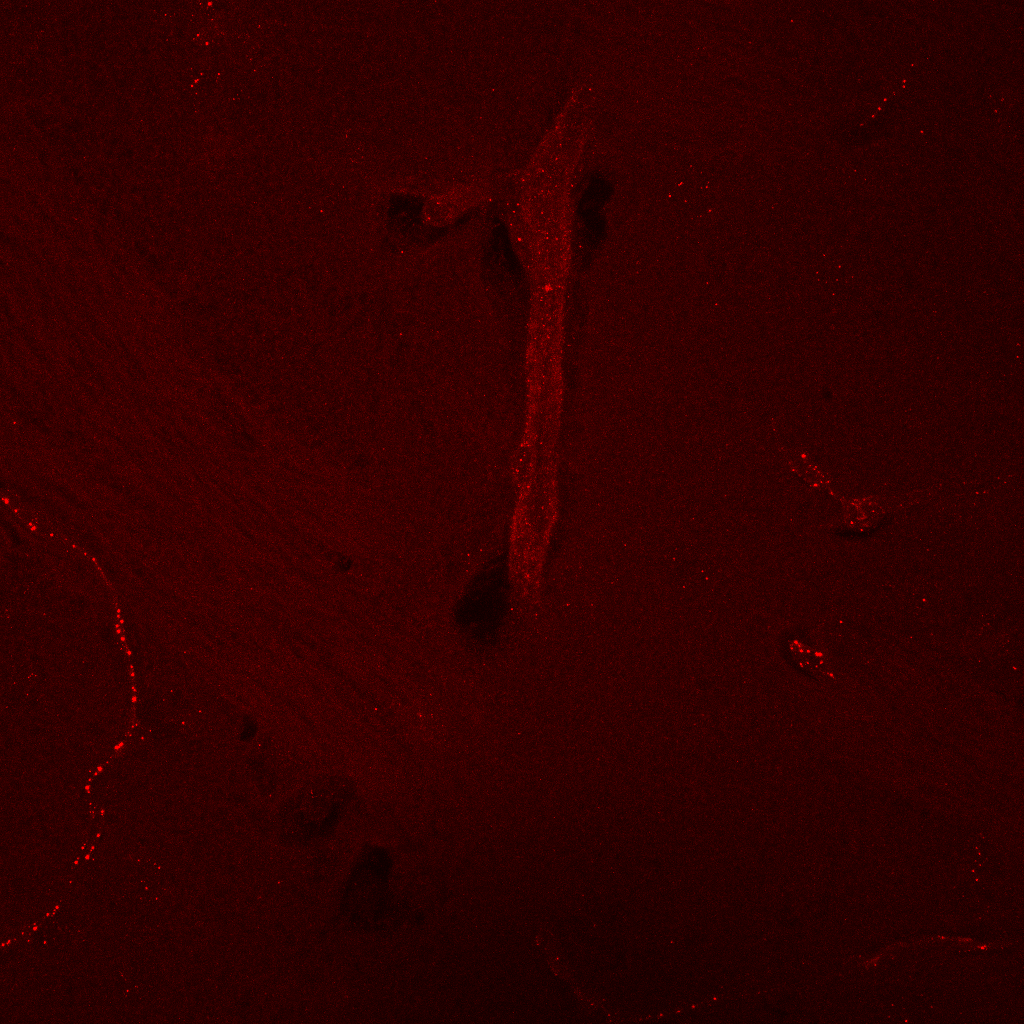

Supplement: Supplementary file 6 — Source Data for Figure 4 [file EMMM-15-e16556-s005.zip › Source data Fig4images 2/7mo_Image1_Asma_TG1_red2.tif]

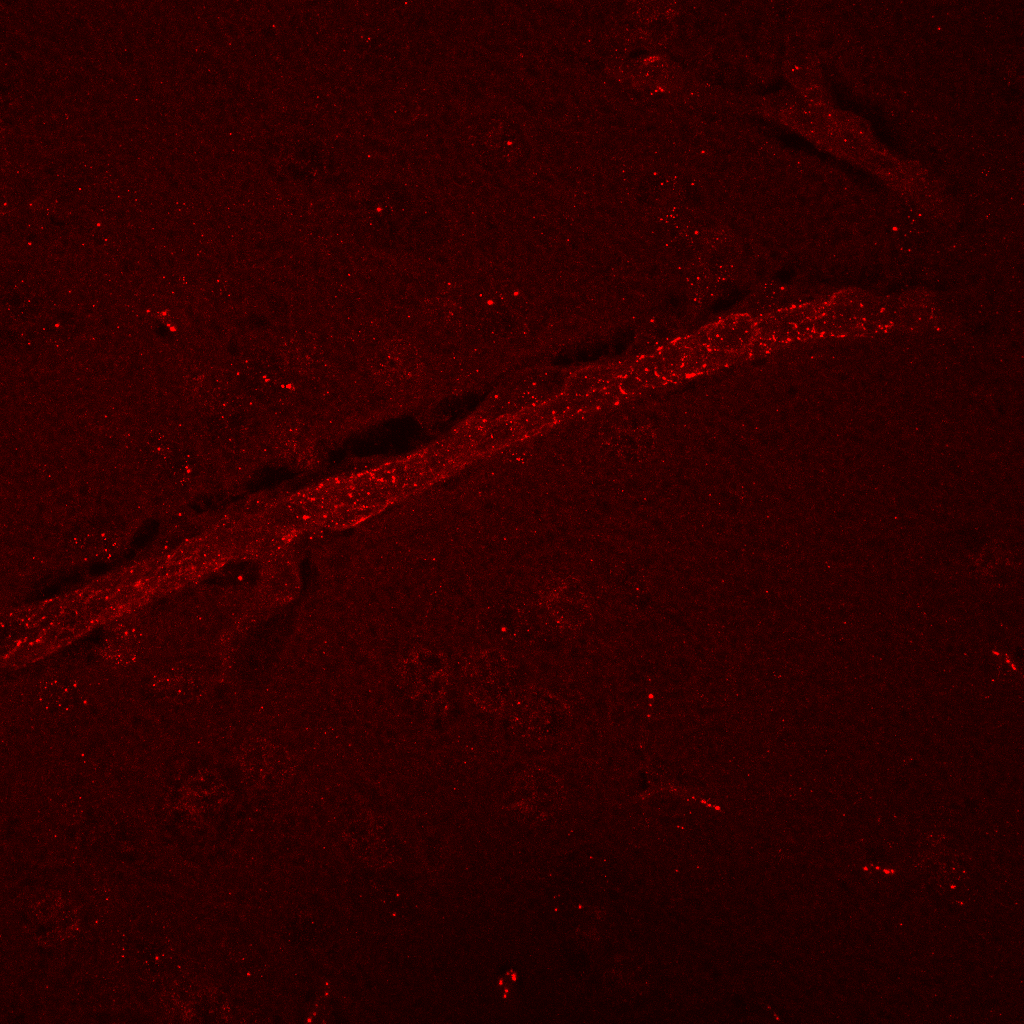

Supplement: Supplementary file 6 — Source Data for Figure 4 [file EMMM-15-e16556-s005.zip › Source data Fig4images 2/18mo_TG18_image5_red.tif]

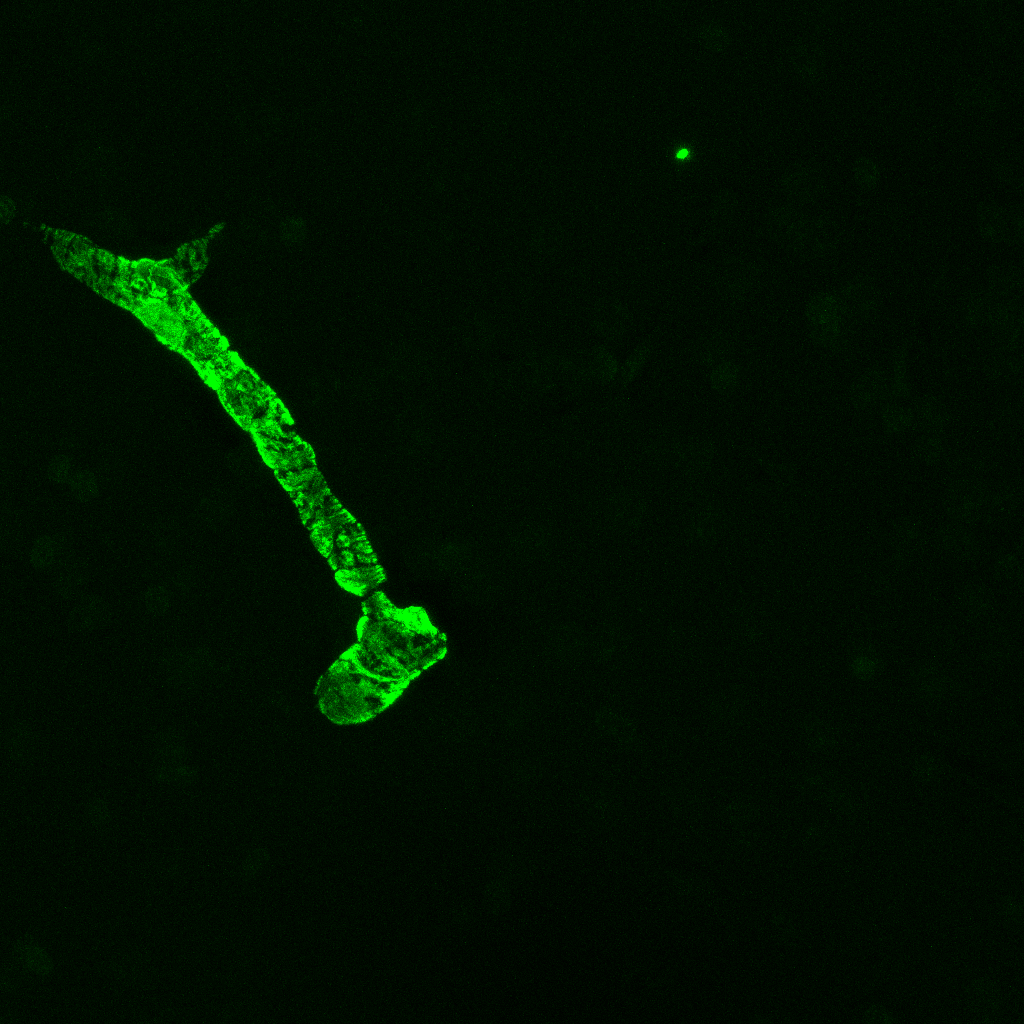

Supplement: Supplementary file 6 — Source Data for Figure 4 [file EMMM-15-e16556-s005.zip › Source data Fig4images 2/Sham_C2_image1_green_final.tif]

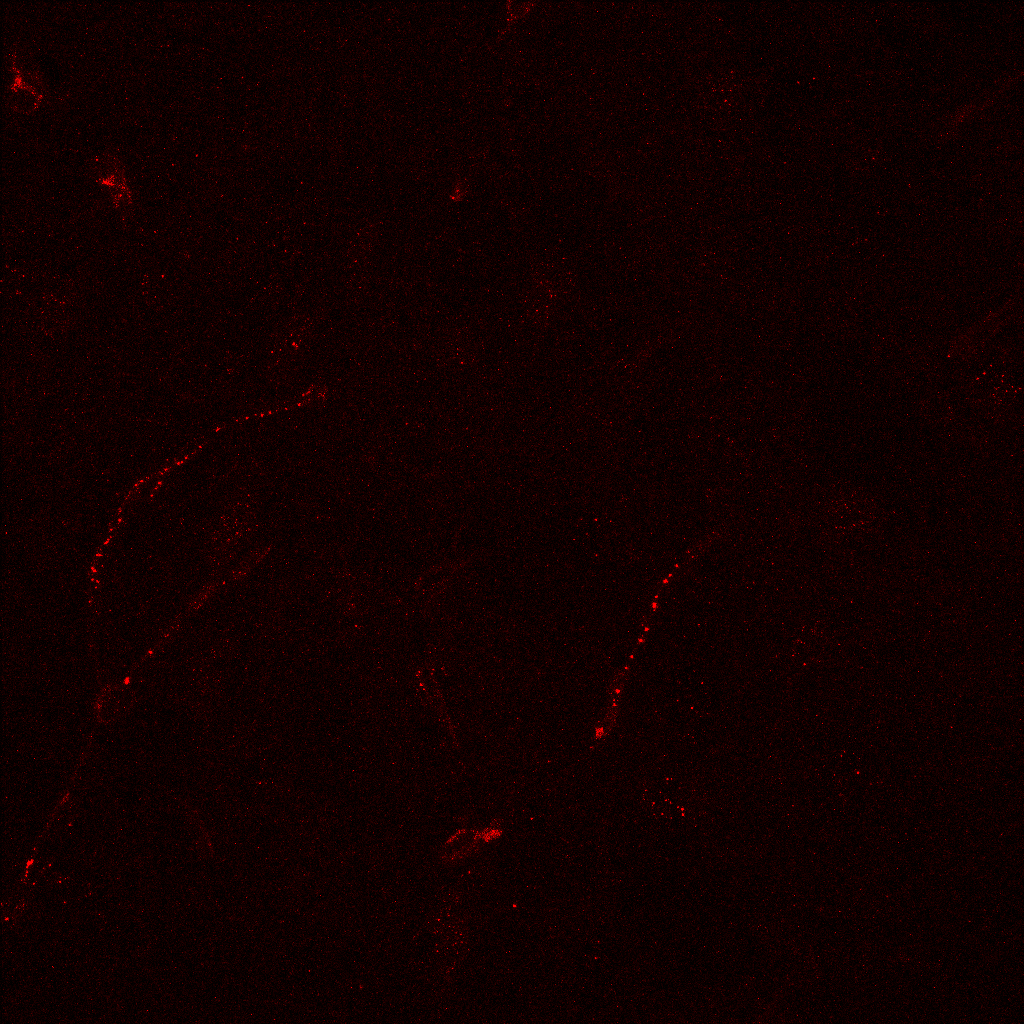

Supplement: Supplementary file 7 — Source Data for Figure 5 [file EMMM-15-e16556-s009.zip › Source data Fig5images/Vaccinated_c2_image1_red.tif]

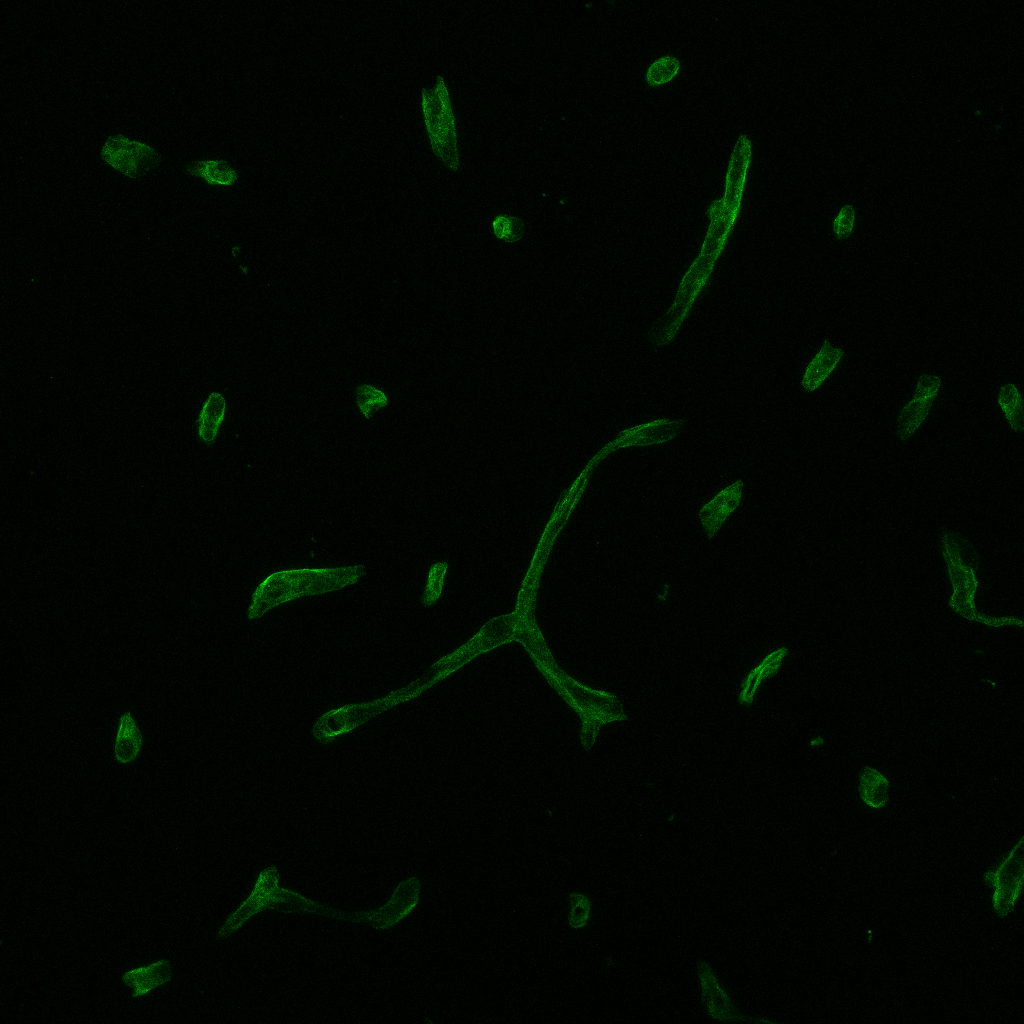

Supplement: Supplementary file 7 — Source Data for Figure 5 [file EMMM-15-e16556-s009.zip › Source data Fig5images/3mo_TG8_image5_green_final.tif]

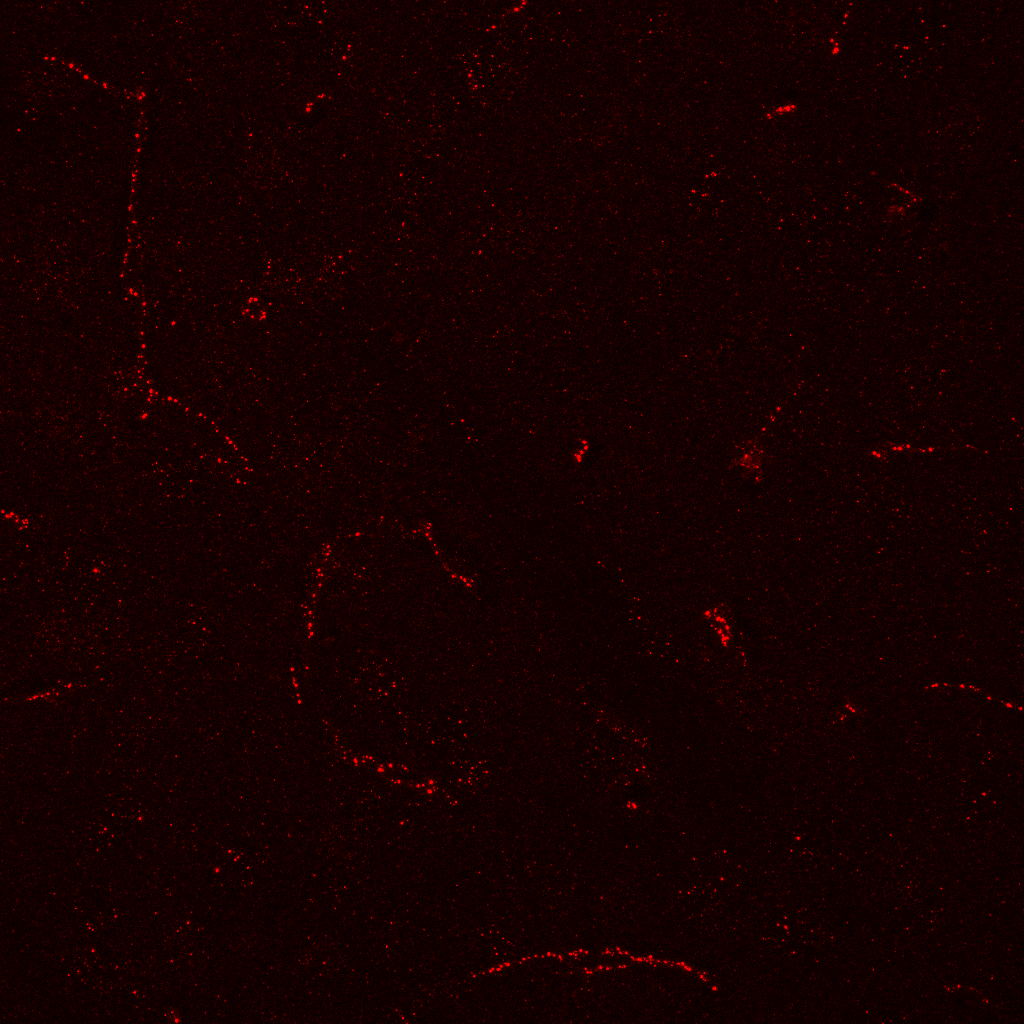

Supplement: Supplementary file 7 — Source Data for Figure 5 [file EMMM-15-e16556-s009.zip › Source data Fig5images/Sham_A6_1min_image1_red1.tiff]

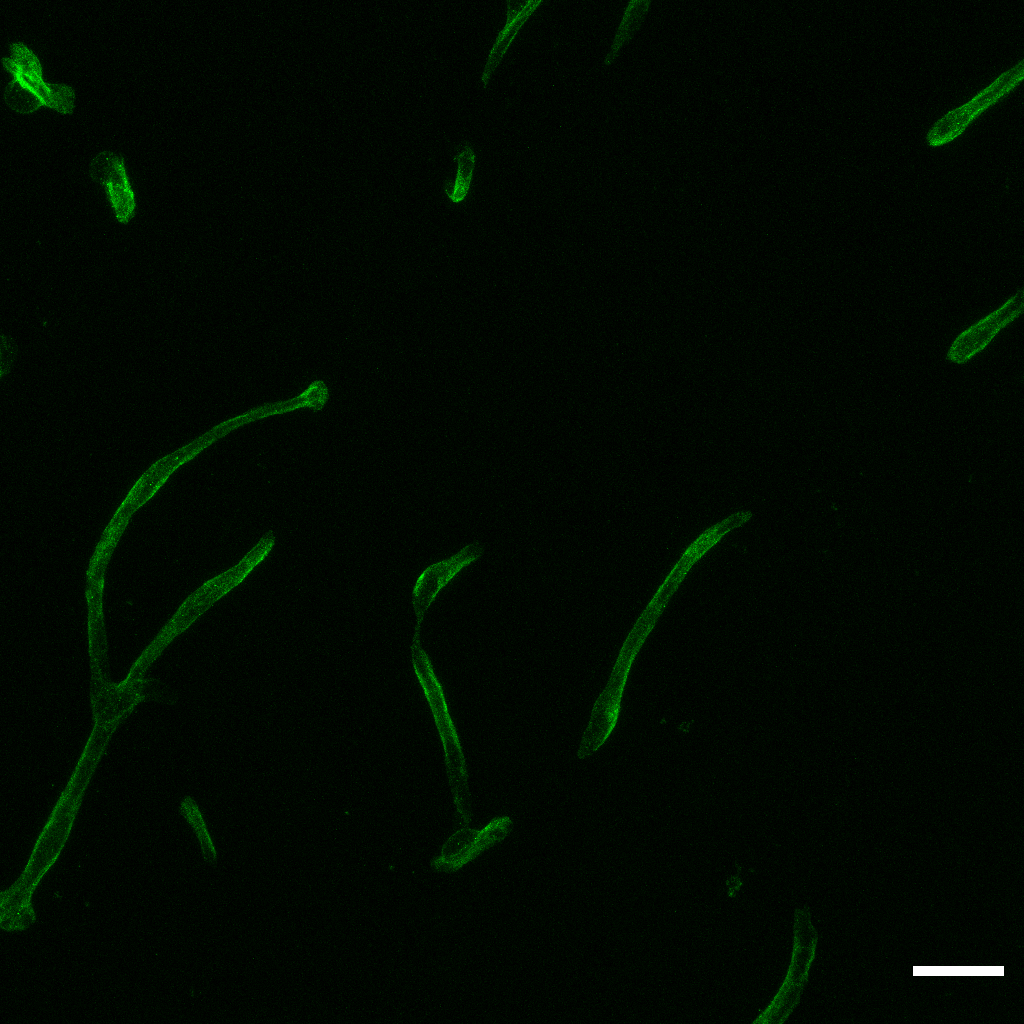

Supplement: Supplementary file 7 — Source Data for Figure 5 [file EMMM-15-e16556-s009.zip › Source data Fig5images/Vaccinated_C2_image1_green.tif]

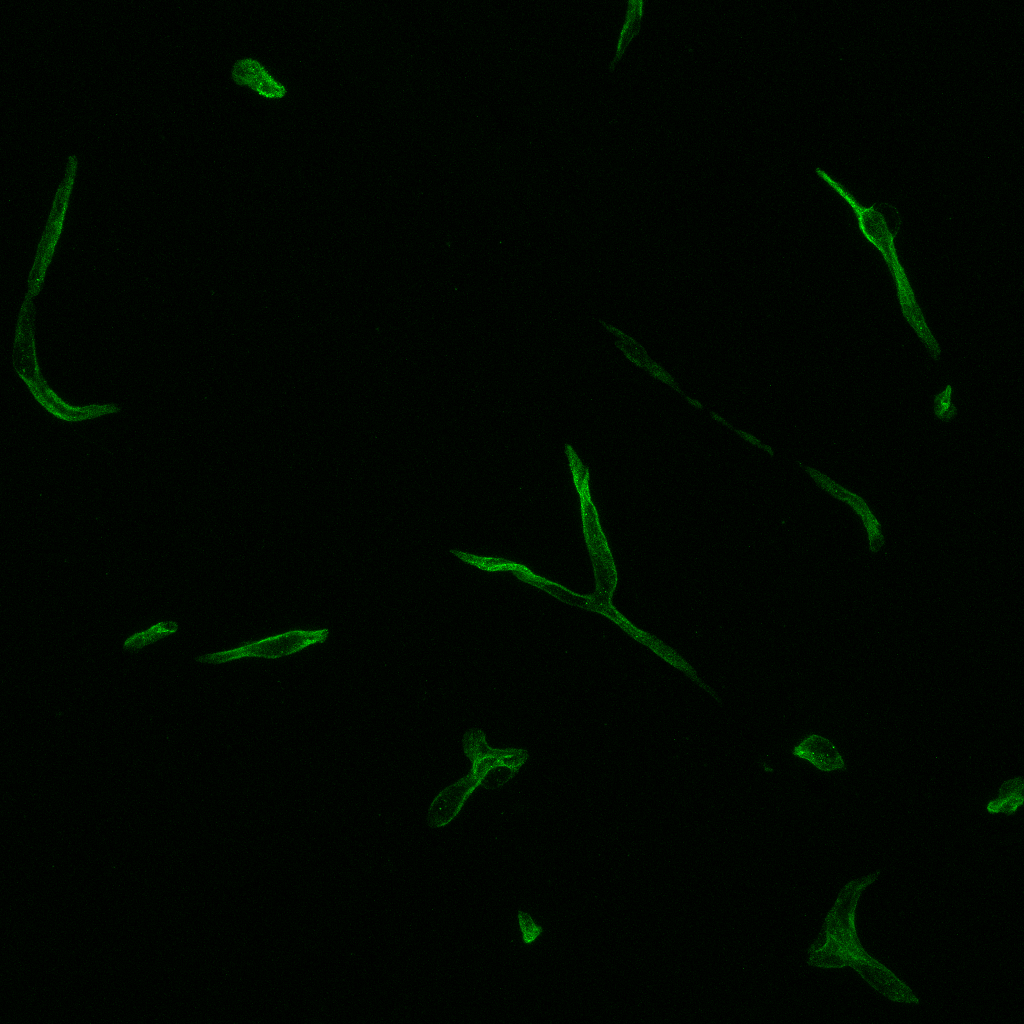

Supplement: Supplementary file 7 — Source Data for Figure 5 [file EMMM-15-e16556-s009.zip › Source data Fig5images/7mo_tg2_MAX_C2-copytg2image4green.tif]

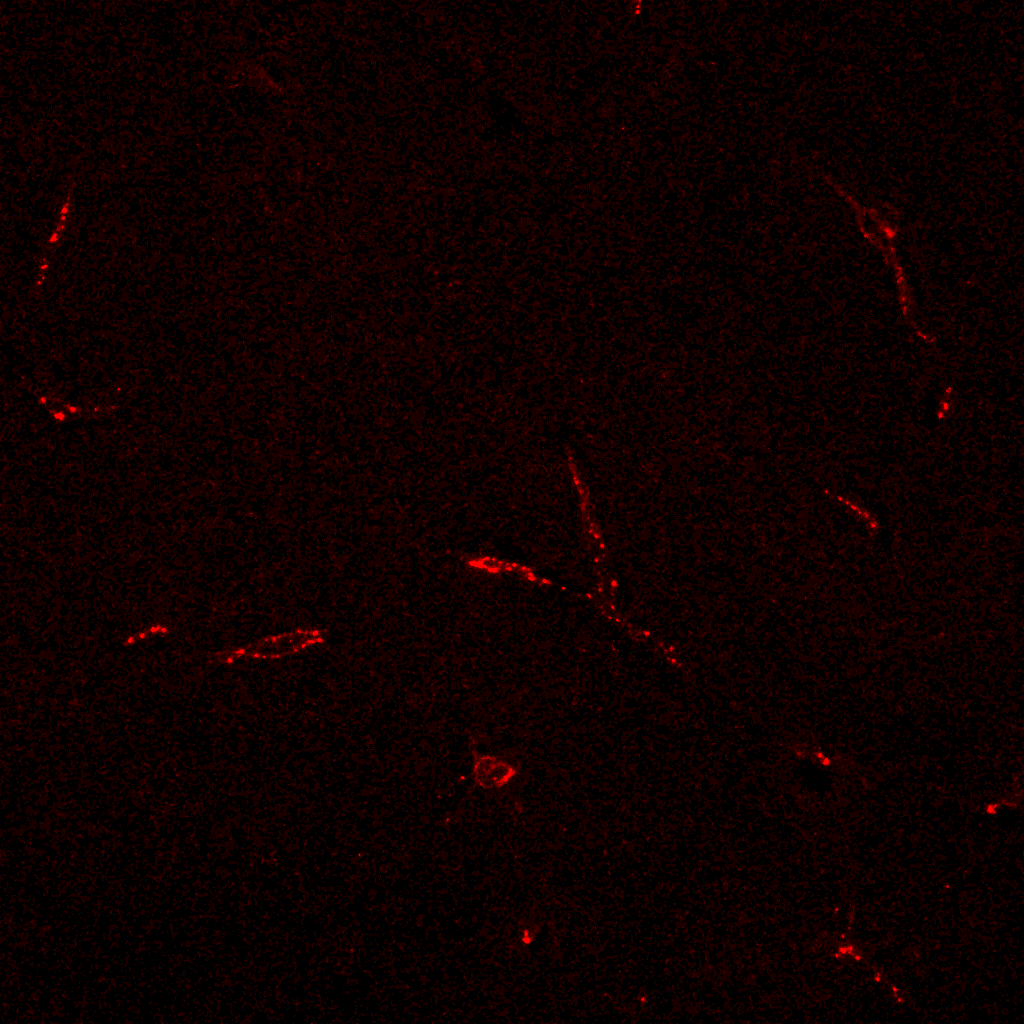

Supplement: Supplementary file 7 — Source Data for Figure 5 [file EMMM-15-e16556-s009.zip › Source data Fig5images/7mo_tg2_MAX_C1-copytg2red-1.tif]

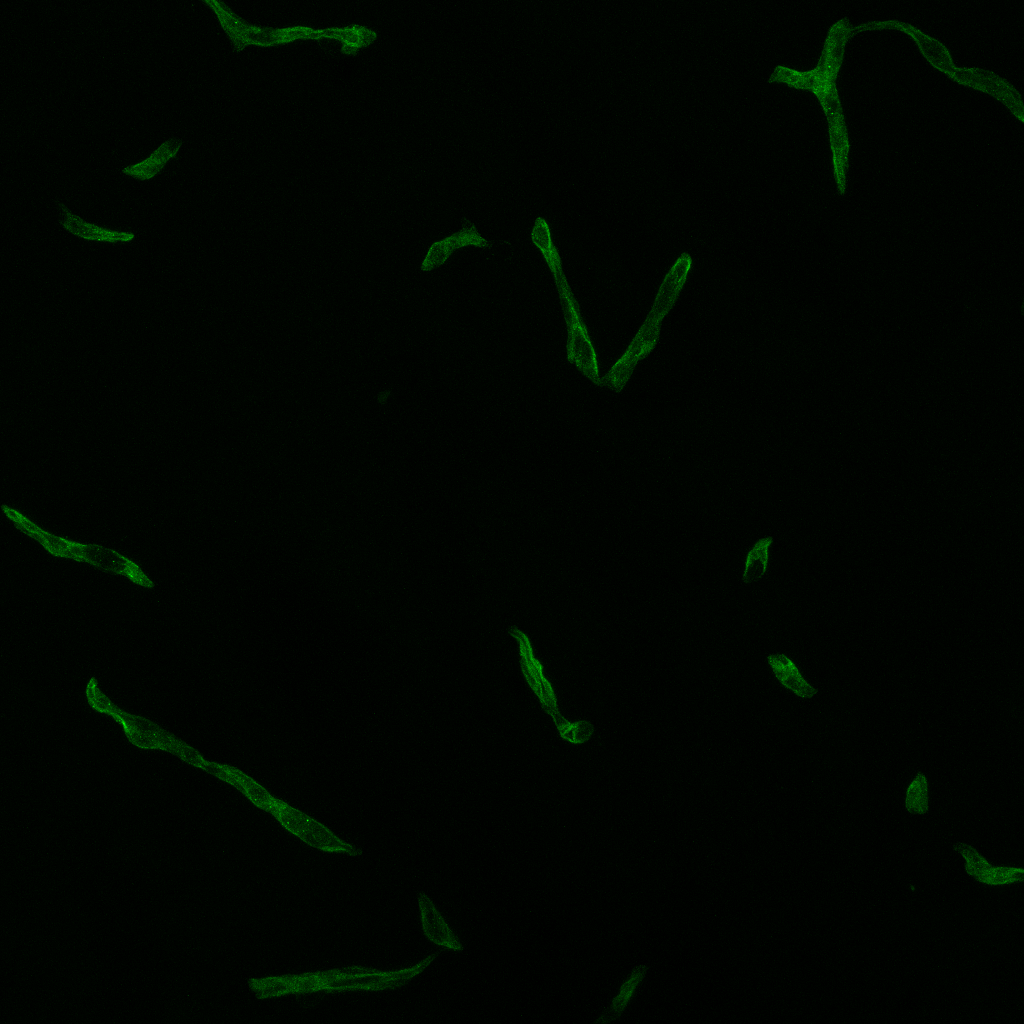

Supplement: Supplementary file 7 — Source Data for Figure 5 [file EMMM-15-e16556-s009.zip › Source data Fig5images/18mo_TG20_15_image9_green.tif]

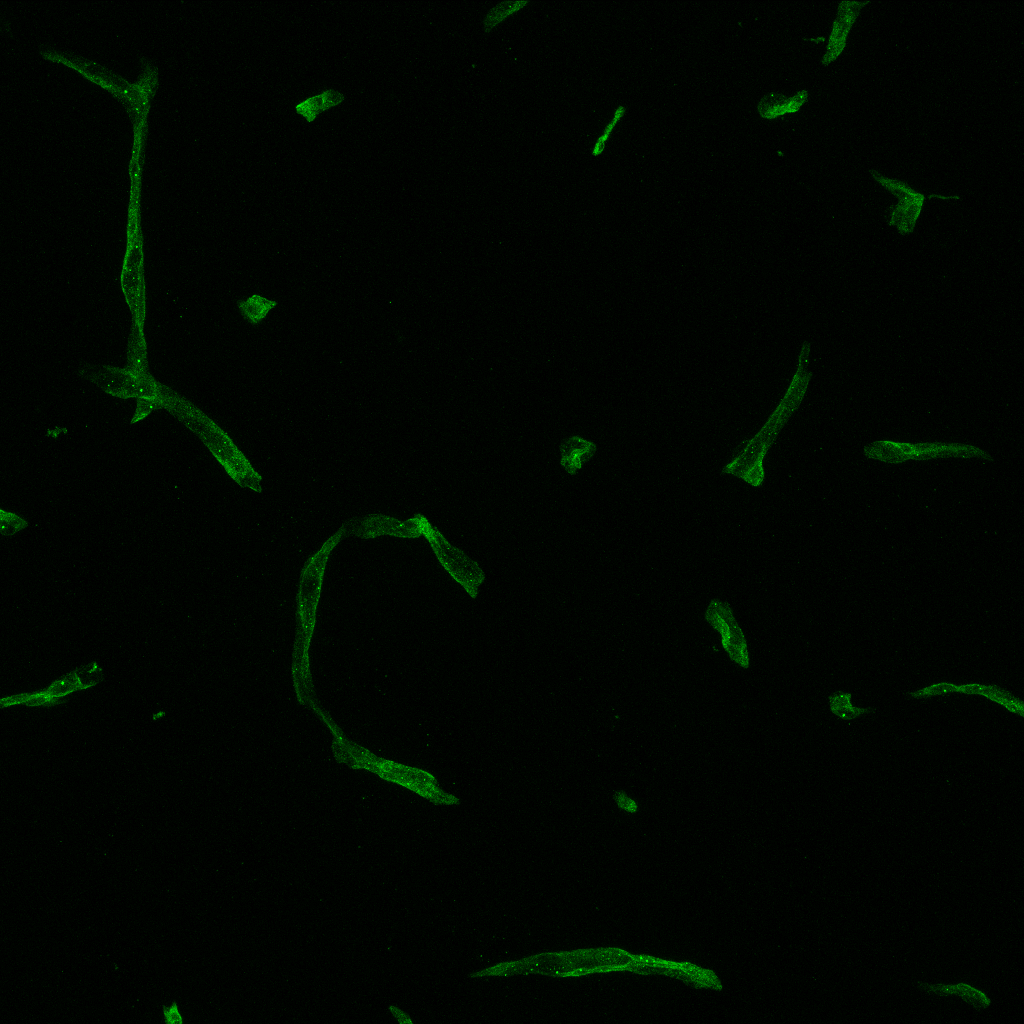

Supplement: Supplementary file 7 — Source Data for Figure 5 [file EMMM-15-e16556-s009.zip › Source data Fig5images/Sham_A6_1min_image1_green.tif]

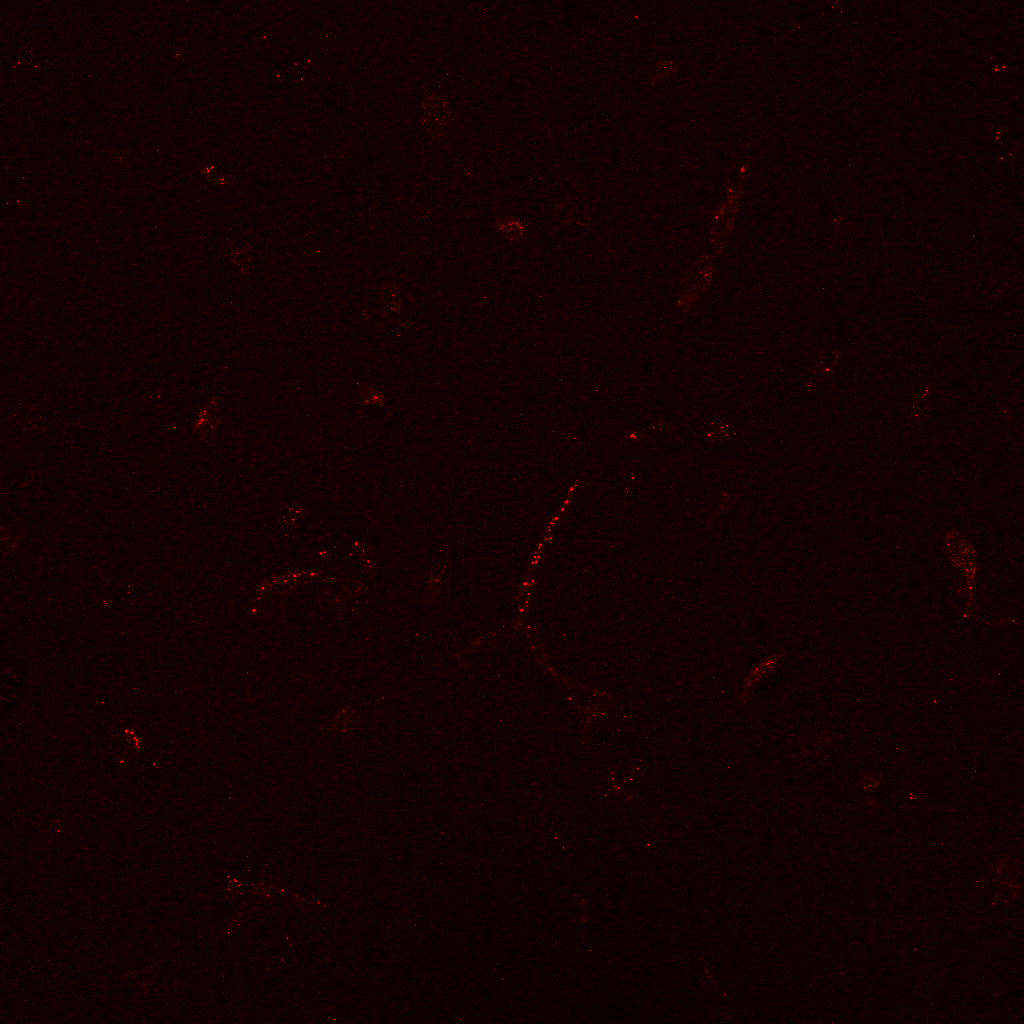

Supplement: Supplementary file 7 — Source Data for Figure 5 [file EMMM-15-e16556-s009.zip › Source data Fig5images/3mo_TG8_image5_red_final.tif]

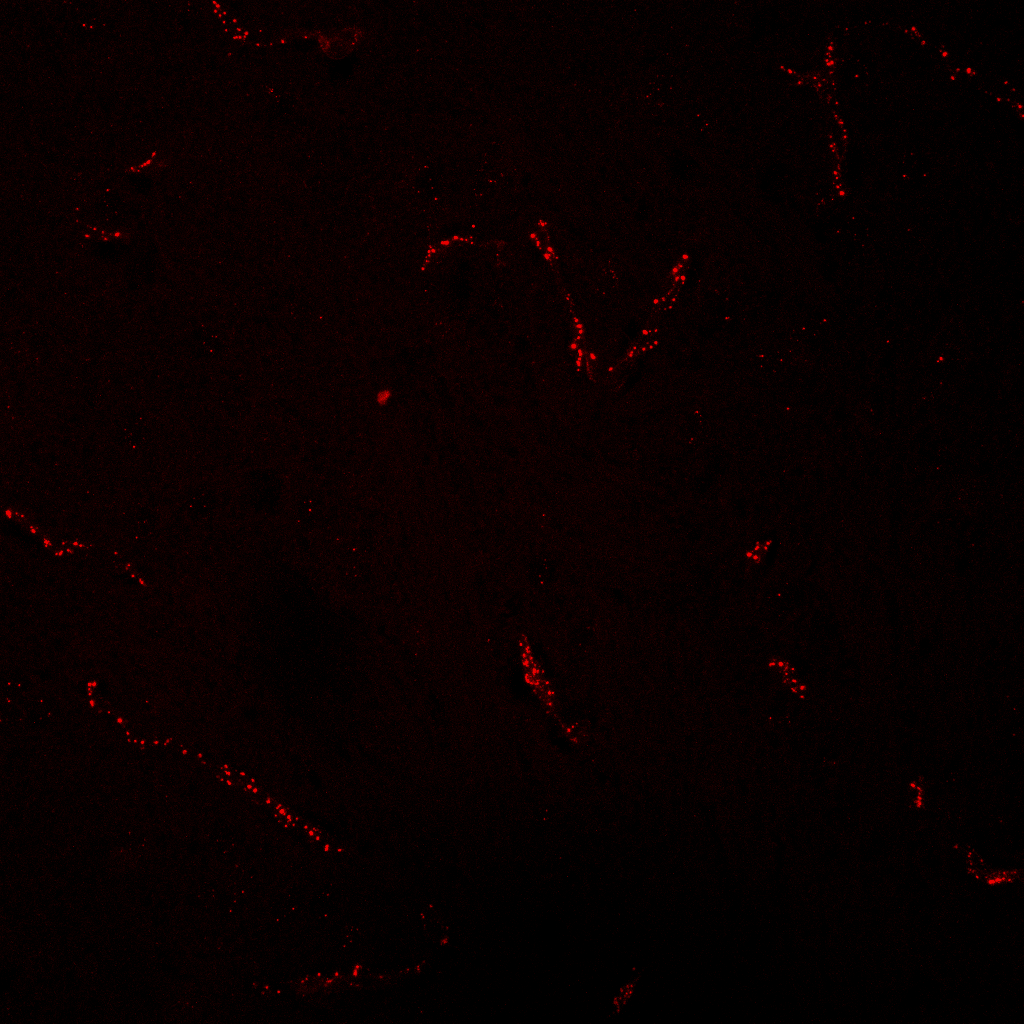

Supplement: Supplementary file 7 — Source Data for Figure 5 [file EMMM-15-e16556-s009.zip › Source data Fig5images/18mo_TG20_15_image9_red.tif]

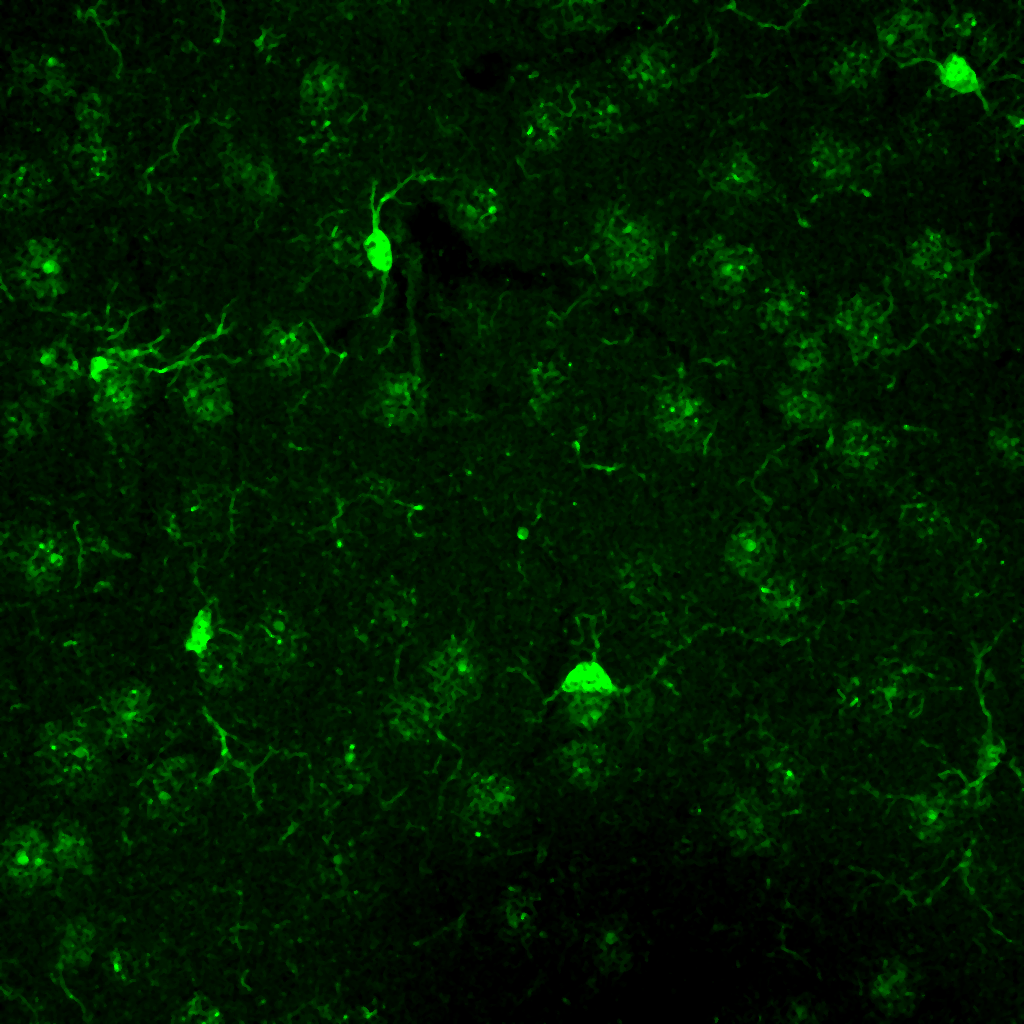

Supplement: Supplementary file 9 — Source Data for Figure 7 [file EMMM-15-e16556-s001.zip › Source data Fig7images/Fig7Cimages/Sham_A3_image13_green.tif]

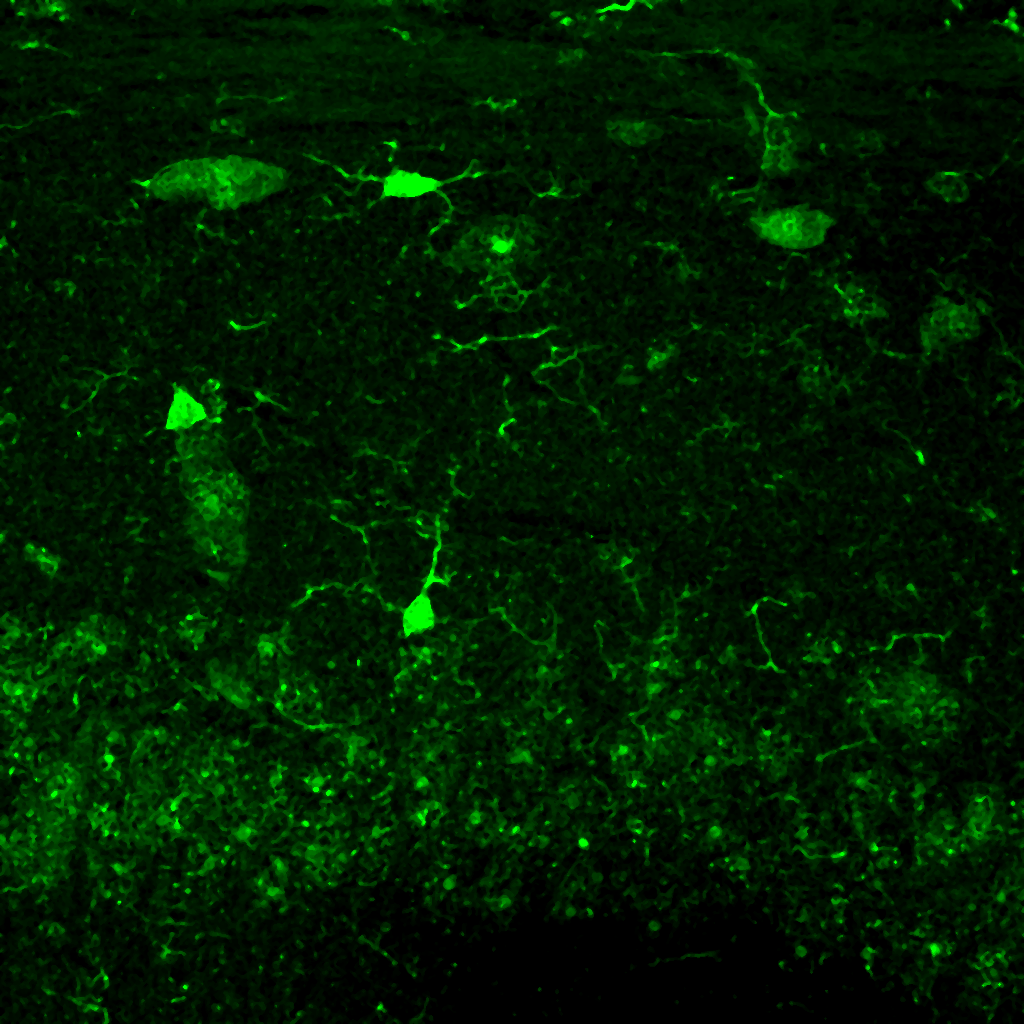

Supplement: Supplementary file 9 — Source Data for Figure 7 [file EMMM-15-e16556-s001.zip › Source data Fig7images/Fig7Cimages/Vaccinated_B1_image4_green.tif]

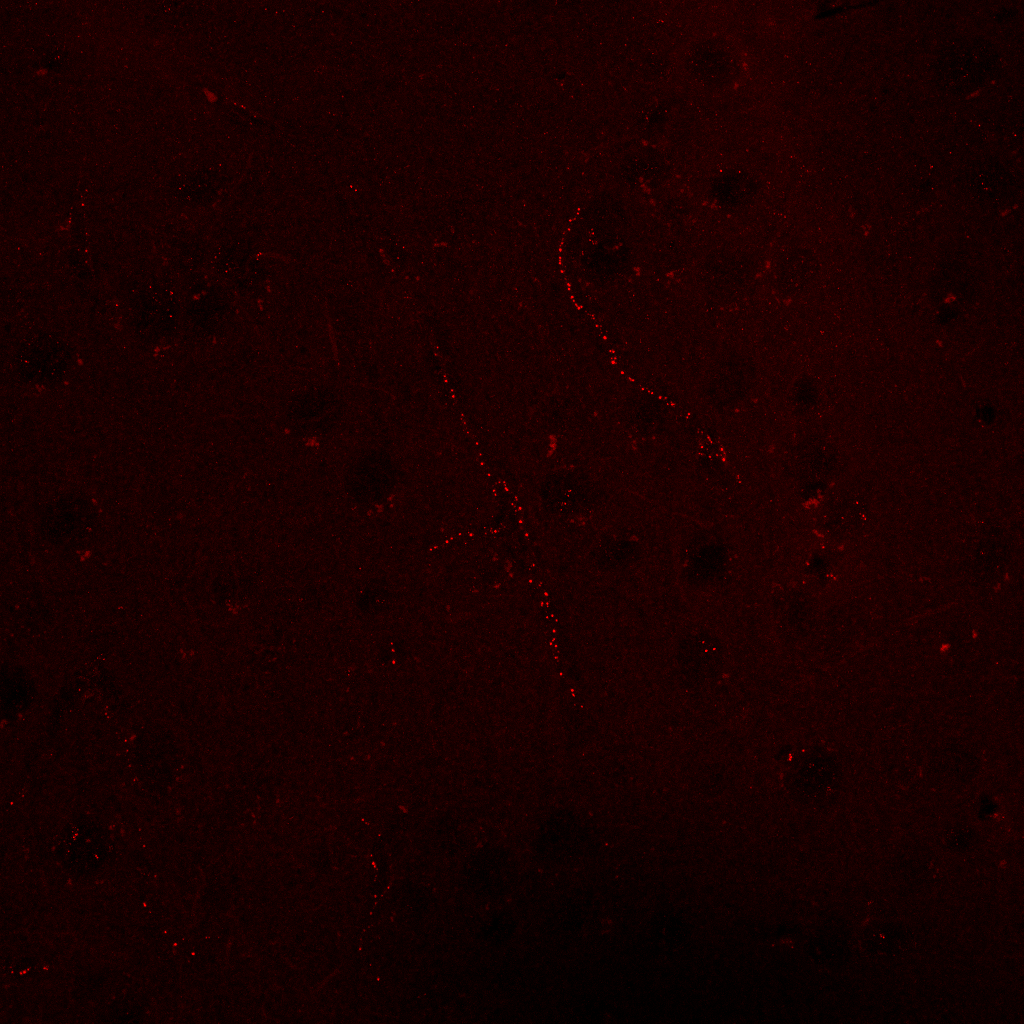

Supplement: Supplementary file 9 — Source Data for Figure 7 [file EMMM-15-e16556-s001.zip › Source data Fig7images/Fig7Cimages/7mo_tg2_image9_red.tif]

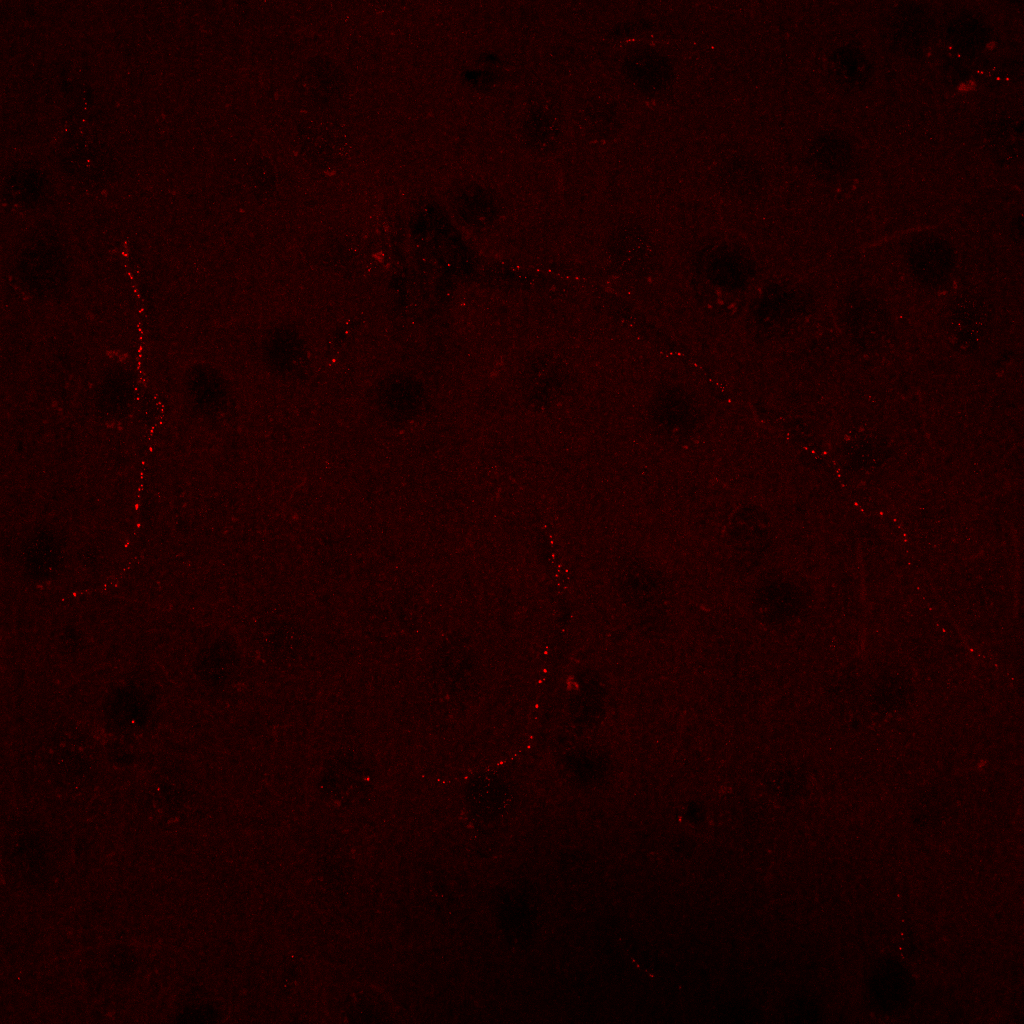

Supplement: Supplementary file 9 — Source Data for Figure 7 [file EMMM-15-e16556-s001.zip › Source data Fig7images/Fig7Cimages/Sham_A3_image13_red.tif]

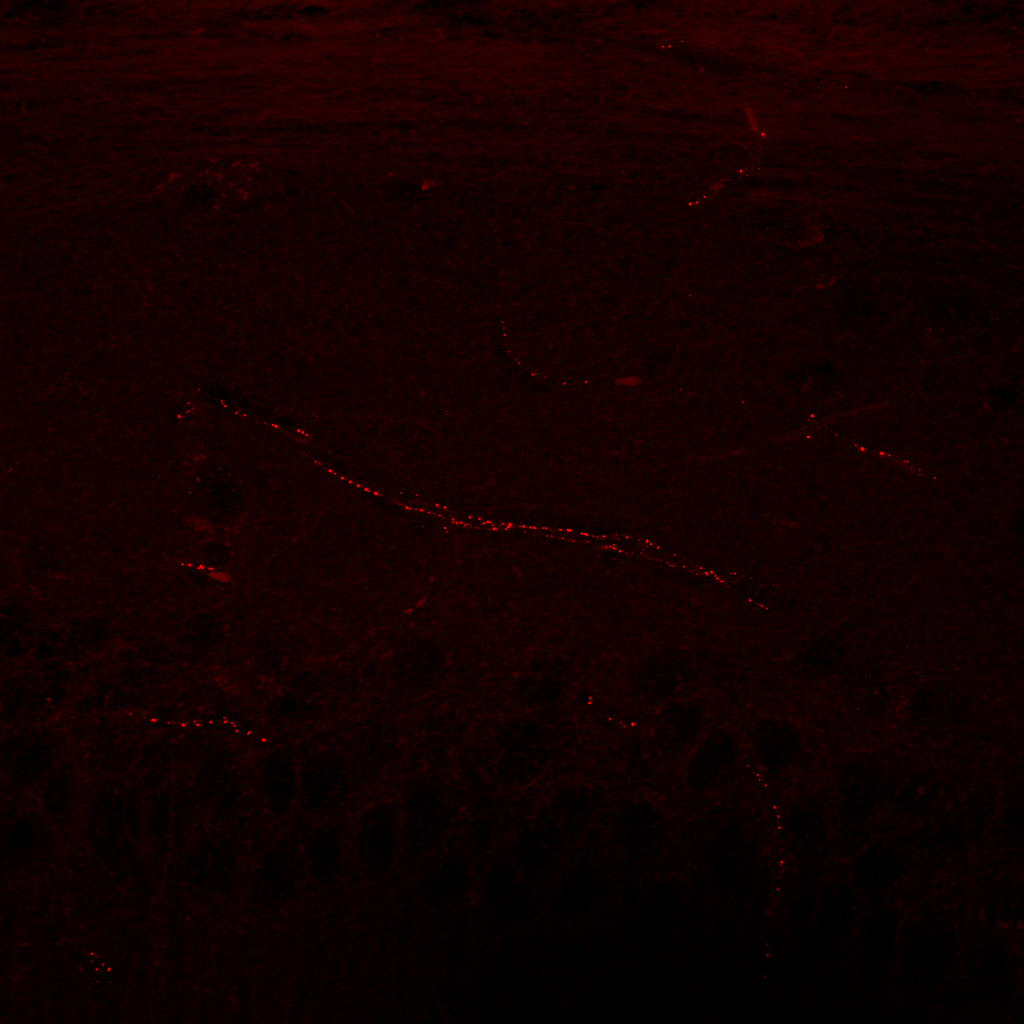

Supplement: Supplementary file 9 — Source Data for Figure 7 [file EMMM-15-e16556-s001.zip › Source data Fig7images/Fig7Cimages/Vaccinated_B1_image4_red.tif]

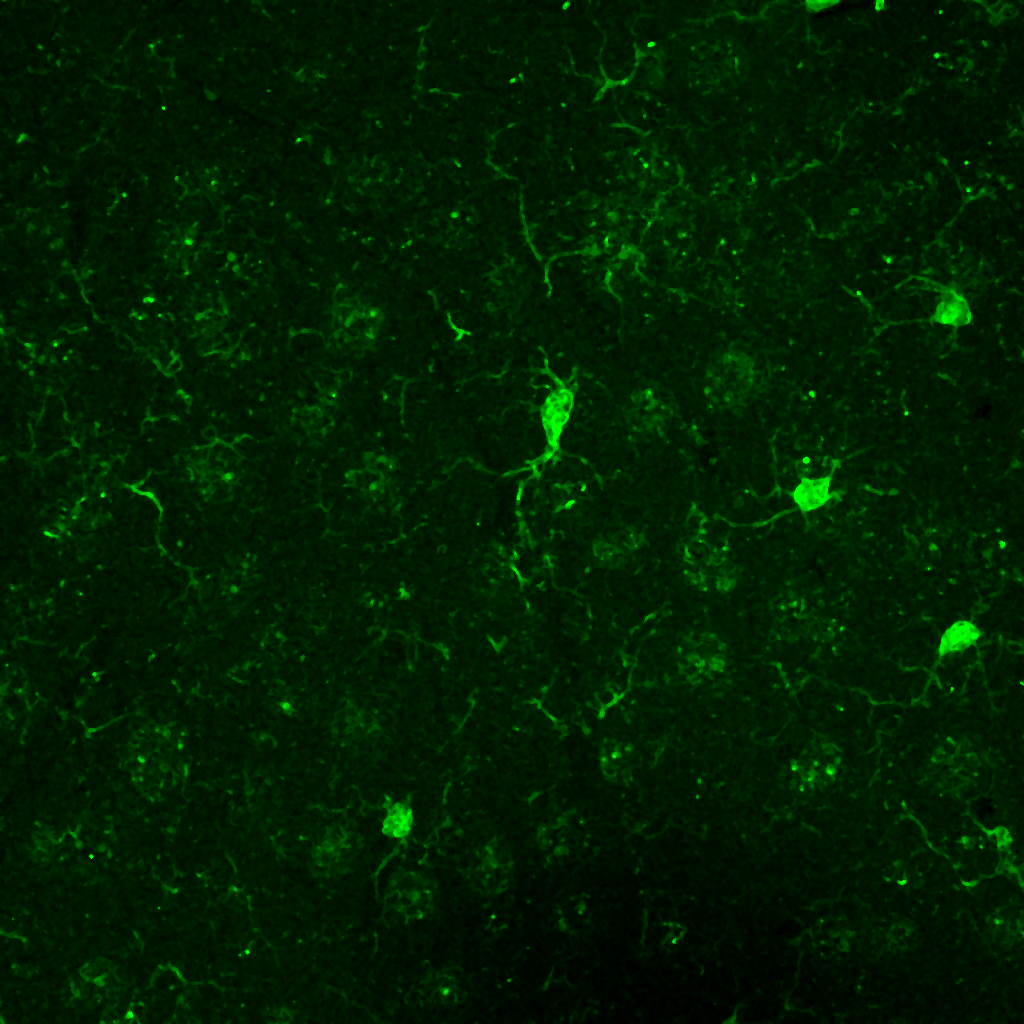

Supplement: Supplementary file 9 — Source Data for Figure 7 [file EMMM-15-e16556-s001.zip › Source data Fig7images/Fig7Cimages/7mo_tg2_image9_green.tif]

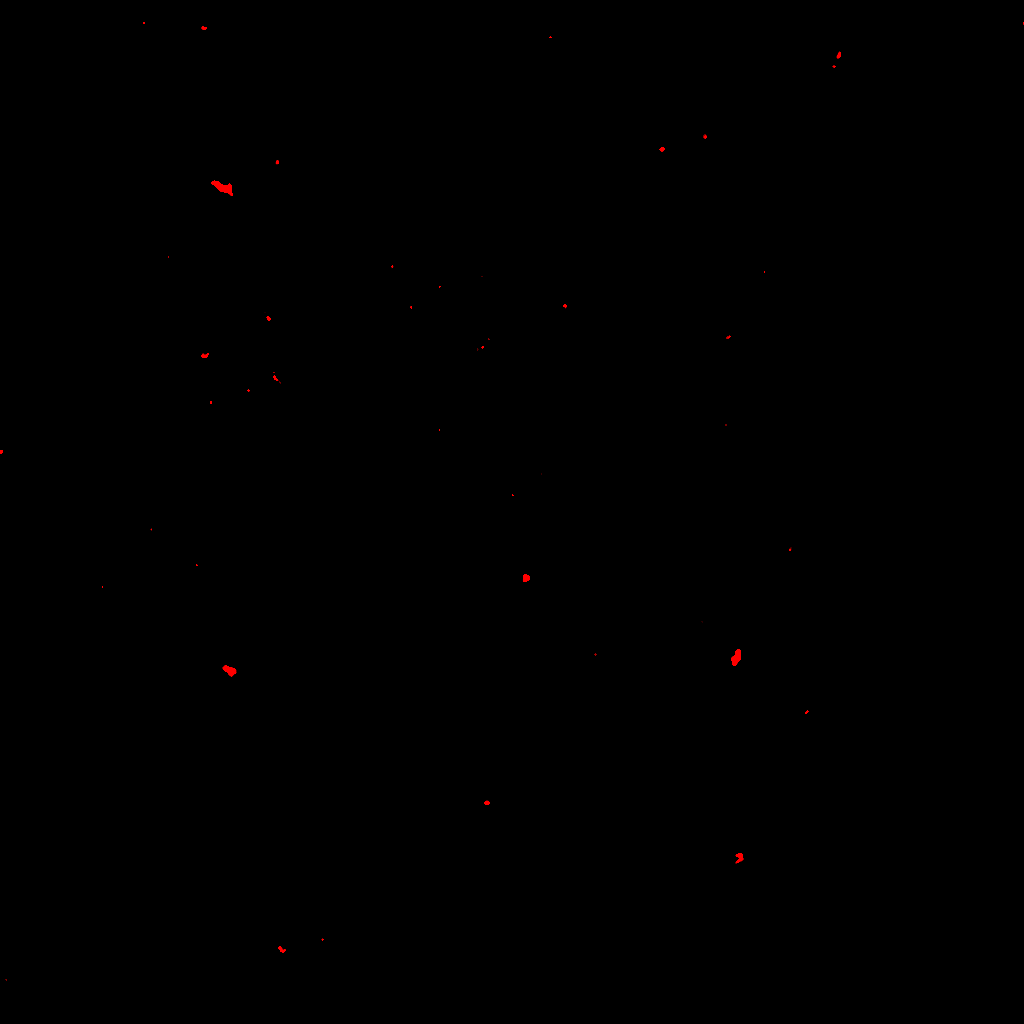

Supplement: Supplementary file 9 — Source Data for Figure 7 [file EMMM-15-e16556-s001.zip › Source data Fig7images/Fig7Aimages/Vaccinated_c1_image40_red.tif]

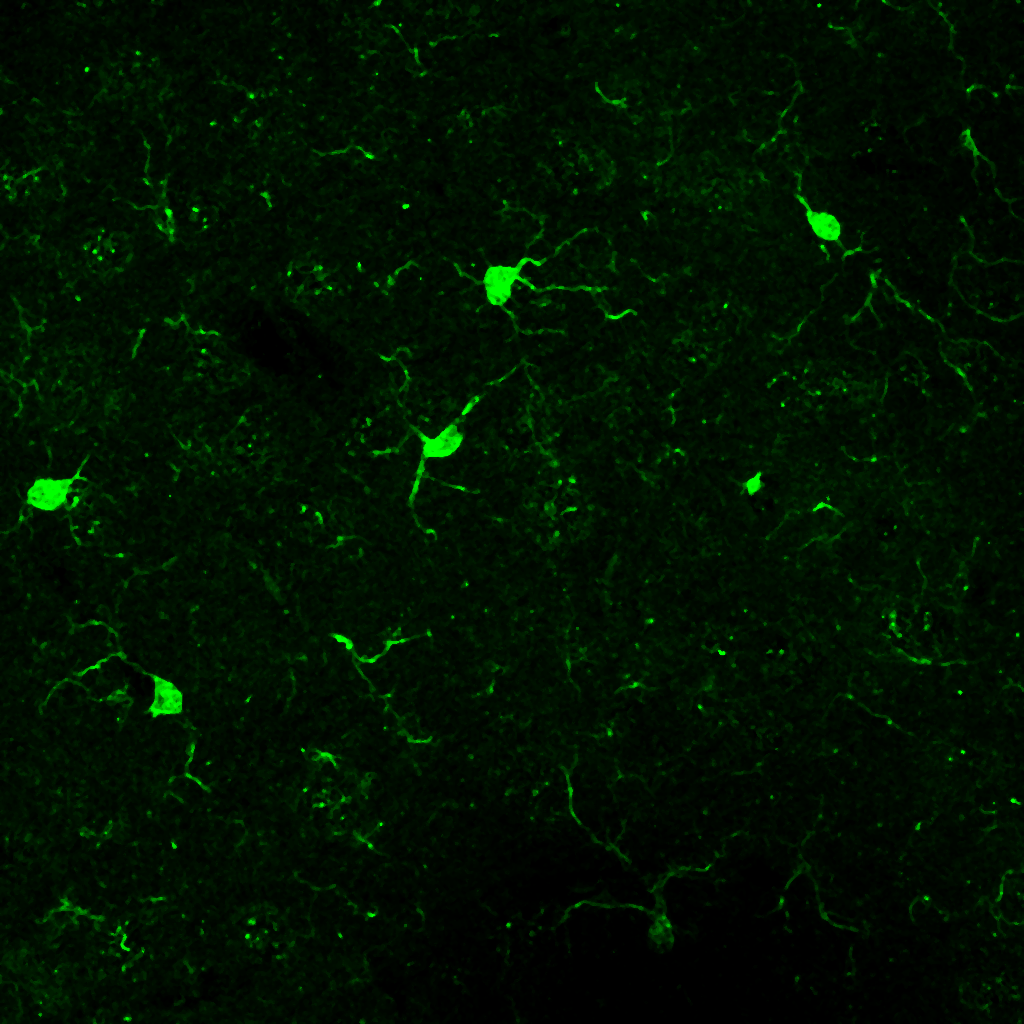

Supplement: Supplementary file 9 — Source Data for Figure 7 [file EMMM-15-e16556-s001.zip › Source data Fig7images/Fig7Aimages/Sham_c4_image_36_green.tif]

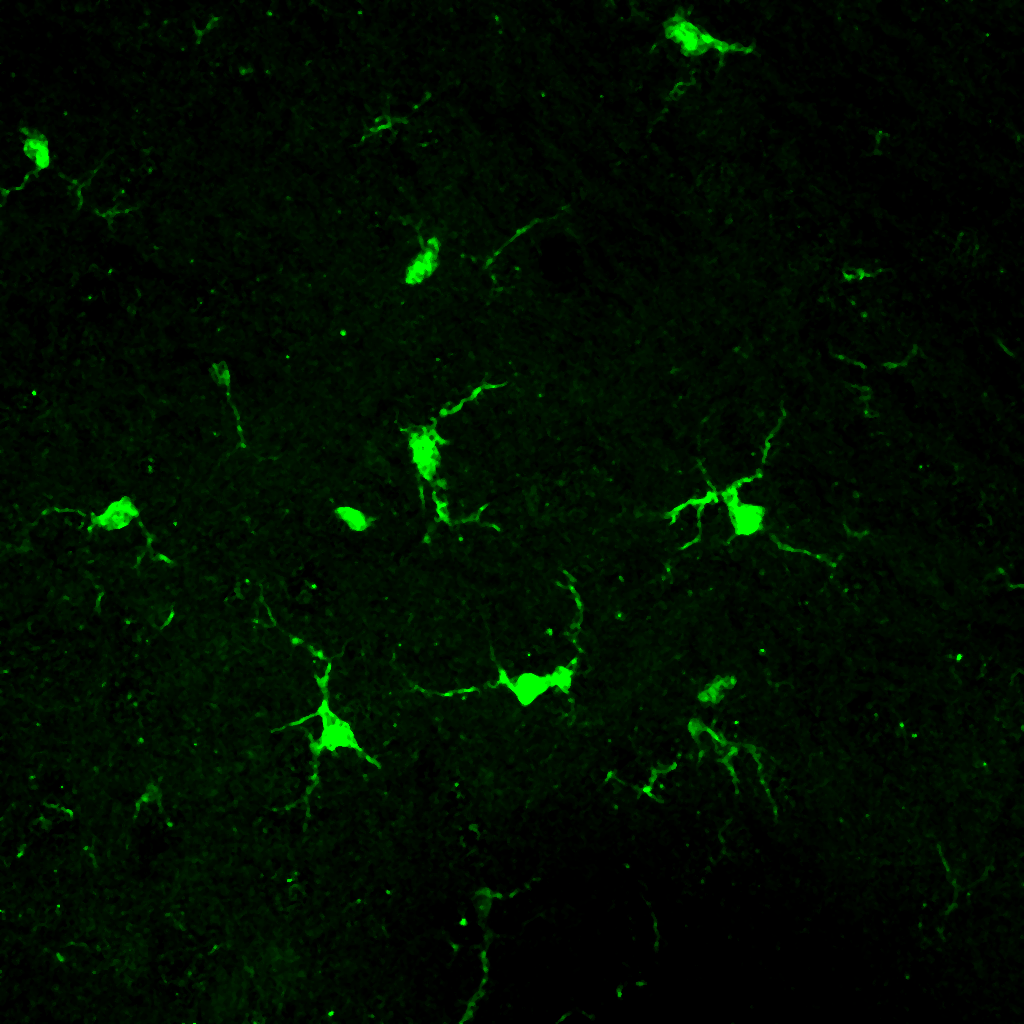

Supplement: Supplementary file 9 — Source Data for Figure 7 [file EMMM-15-e16556-s001.zip › Source data Fig7images/Fig7Aimages/7mo_tg4_image12_green.tif]

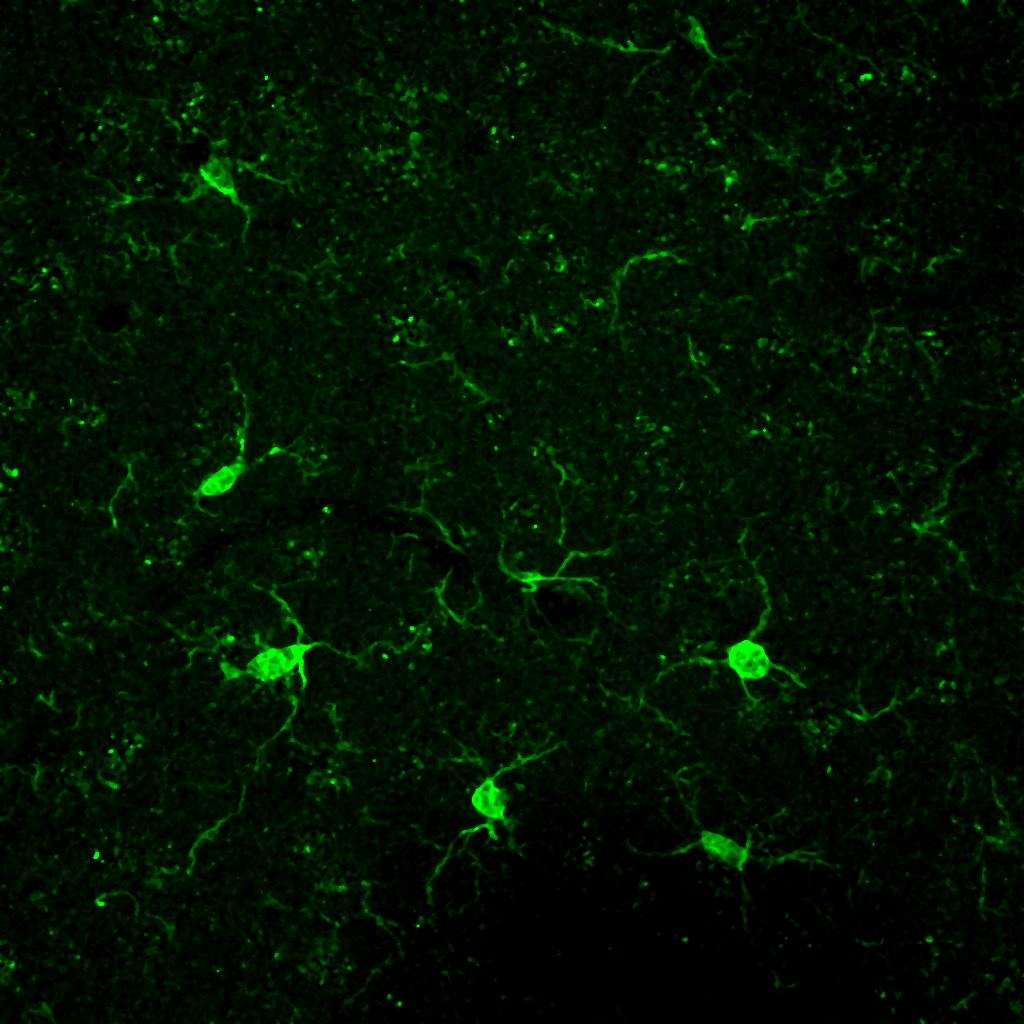

Supplement: Supplementary file 9 — Source Data for Figure 7 [file EMMM-15-e16556-s001.zip › Source data Fig7images/Fig7Aimages/Vaccinated_c1_image40_green.tif]

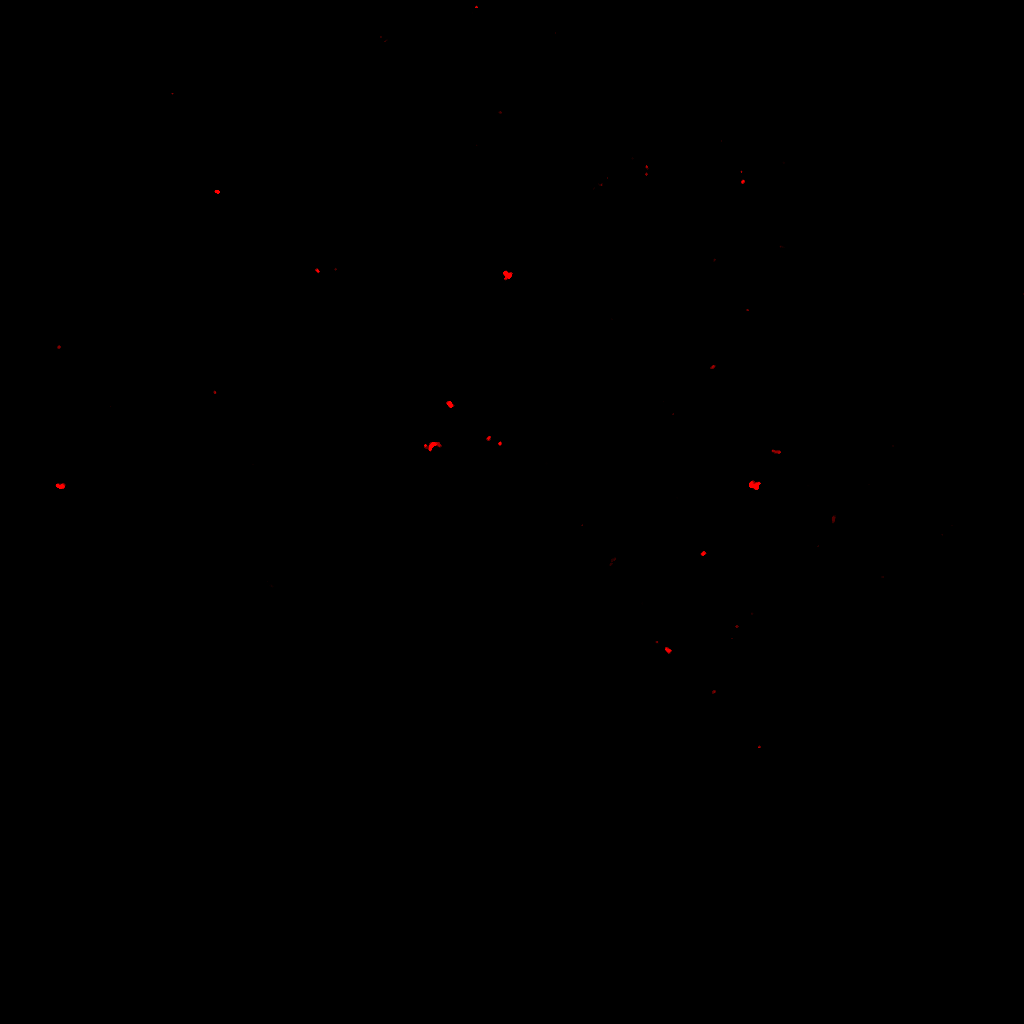

Supplement: Supplementary file 9 — Source Data for Figure 7 [file EMMM-15-e16556-s001.zip › Source data Fig7images/Fig7Aimages/Sham_c4_image_image36_red.tif]

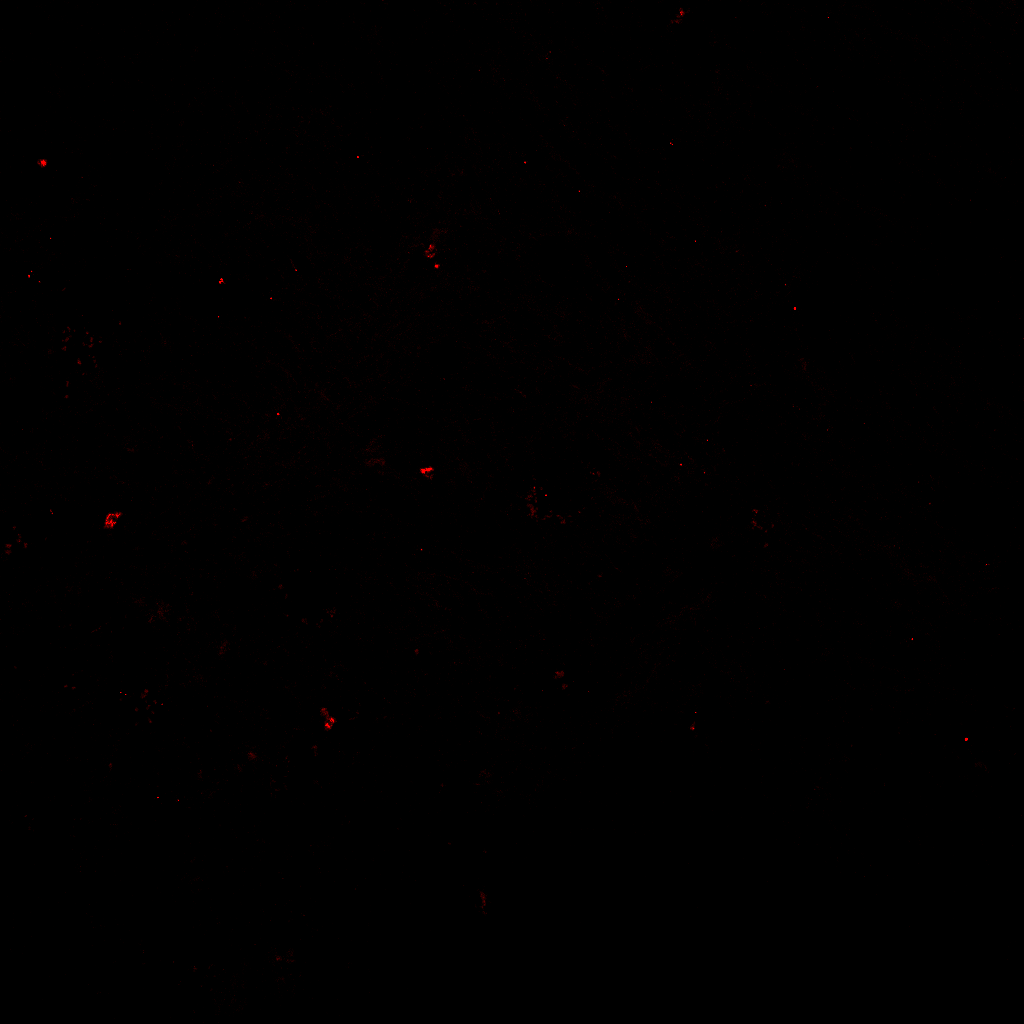

Supplement: Supplementary file 9 — Source Data for Figure 7 [file EMMM-15-e16556-s001.zip › Source data Fig7images/Fig7Aimages/7mo_tg4_image12_red.tif]
