# Supplementary material for: Active immunotherapy reduces NOTCH3 deposition in brain capillaries in a CADASIL mouse model
Source: EMBO Mol Med. 2022 Dec 16;15(2):e16556. doi: 10.15252/emmm.202216556 (PMC9906330; doi:10.15252/emmm.202216556)
Supplement: Supplementary file 10 [file EMMM-15-e16556-s006.pdf]

EMBO Molecular Medicine

Active immunotherapy reduces NOTCH3 deposition in brain capillaries in a CADASIL mouse model

Daniel Oliveira, Kirsten Coupland, Wenchao Shao, shaobo jin, Francesca Del Gaudio, Sailan Wang, Rhys Fox, Julie Rutten, Johan Sandin, Henrik Zetterberg, Johan Lundkvist, Saskia Lesnik Oberstein, Urban Lendahl, and Helena Karlstrom
DOI: 10.15252/emmm.202216556

Corresponding authors: Helena Karlstrom (helena.karlstrom@ki.se) , Urban Lendahl (Urban.Lendahl@ki.se)

Review Timeline:

Transfer from Review Commons:	8th Jul 22
Editorial Decision:	12th Jul 22
Revision Received:	4th Oct 22
Editorial Decision:	25th Oct 22
Revision Received:	14th Nov 22
Accepted:	28th Nov 22

Editor: Lise Roth

Transaction Report:

This manuscript was transferred to EMBO Molecular Medicine following peer review at Review Commons.

(Note: With the exception of the correction of typographical or spelling errors that could be a source of ambiguity, letters and reports are not edited. Depending on transfer agreements, referee reports obtained elsewhere may or may not be included in this compilation. Referee reports are anonymous unless the Referee chooses to sign their reports.)

Review #1

1. Evidence, reproducibility and clarity:

Evidence, reproducibility and clarity (Required)

Summary:

This study have used immunogenic aggregates formed by recombinant Notch3 fragments EGF 1-5 that contain CADASIL NOTCH3 R133C and wild type NOTCH3 to inoculate a CADASIL mouse model TgN3R182C as an active immunisation therapy of CADASIL. The vaccinated mice showed a decreased deposition of NOTCH3 around brain capillaries, reduced blood NOTCH3 ECD and microglia activation, suggesting a potential novel therapy for future treatment of CADASIL. The lack of impact on retinal vasculature, body weight, and general behaviour of the treated mice indicate the safety of the therapy.

Major comments:

The results are interesting and the manuscript was carefully written and presented. However, the reduction of NOTCH3 in blood samples and the deposition around capillaries were so modest, plus there was no significant change of NOTCH3 deposition in arterioles. This questions the real effectiveness of this immunisation therapy when translating to clinical patients.

Minor comments:

- Figure EV1 is redundant as Figure 2C has the same information.
- P5 last line "there was a prominent loss of monomeric NOTCH3 EGF1-5 when WT and R133C fragments were mixed as compared to incubating them separately (Fig 2C)". Why the aggregation is more obvious when mutant protein fragment mixed with wild type comparing mutant fragment alone?
- How was the dosage or concentration of aggregated protein (0.5 mg/ml) used for immunisation determined?
- P7 line 1-2, while using ASMA as a marker for arteries/arterioles, didn't the author see any expression of ASMA in pericytes (capillaries)?
- In Figure 5A, the NOTCH3 ECD signal looks like similar between Shame and Vaccinated, although the quantifications seem significant in Figure 5B. The author may discuss the robustness of the quantitation method used and therefore the conclusion (i.e., active immunization with NOTCH3 EGF1-5 WT/R133C aggregates specifically reduces the amount of NOTCH3 aggregates around cerebral capillaries.).
- In Figure 8B, what does the "% of microglia area" mean and how was it calculated?
- Figure 9A doesn't seem to support the conclusion "There was also a trend towards more NOTCH3 ECD deposits inside or in close vicinity to microglia in vaccinated TgN3R182C150 mice compared to control or sham-vaccinated TgN3R182C150 mice, although the difference did not reach statistical significance", as it is not so convincing that the signals of the NOTCH3 ECD staining co-localise with microglia in the vaccinated

sample. Besides, what's the meaning or functional significance about "% of microglia area", "number/1000 um2 microglia", and "average size per microglia"?

2. Significance:

Significance (Required)

CADASIL is the most common genetic small vessel disease that leads to cognitive defect and eventual vascular dementia. Current, there is no specific treatment available. Similar to a number of neurodegenerative conditions caused by protein accumulations like Alzheimer's disease, Parkinson disease and Huntington disease etc, NOTCH3 protein accumulation represents a key pathological change in CADASIL and therefore a drug target. The approach of active immunisation therapy described in this paper demonstrated a novel method for the treatment of this condition. Although the effectiveness of the therapy in the transgenic mouse model of CADASIL was yet highly impressive, this paper provides a proof-of-principle that the active immunisation is more or less functional and, most importantly, tolerable. One other advantage of this approach is that the immunisation therapy is not restricted to a specific NOTCH3 mutation. After further development this strategy could potentially benefit patients in the future.

This paper may be interested by researchers working on diseases that are caused by specific protein accumulation or aggregations.

My expertise is in the area of studying the molecular mechanisms of CADASIL and other genetic small vessel diseases.

3. How much time do you estimate the authors will need to complete the suggested revisions:

Estimated time to Complete Revisions (Required)

(Decision Recommendation)

Less than 1 month

4. Review Commons values the work of reviewers and encourages them to get credit for their work. Select 'Yes' below to register your reviewing activity at Publons; note that the content of your review will not be visible on Publons.

Reviewer Publons

Yes

Review #2

1. Evidence, reproducibility and clarity:

Evidence, reproducibility and clarity (Required)

The article submitted by Daniel V. Oliveira et al to Review Commons and titled "NOTCH3 active immunotherapy reduces NOTCH3 deposition in brain capillaries in a CADASIL mouse model" describes a novel active immunization therapy against the aggregated NOTCH3 mutant protein associated to CADASIL pathology. This strategy induces a significant reduction in NOTCH3 deposition around brain capillaries, increase of microglia activation and lowering of serum levels of NOTCH3, that demonstrates the potential clinical value of the therapy. In addition, the authors report that the therapy is safe and tolerable. The study is well written, very clear and the results are very promising.

Same minor comments need to be clarified before publication.

1. In the introduction is indicated that ".....Aducanumab recently being approved for treatment of AD by the Food and Drug Association (Budd Haeberlein et al, 2017; Mintun et al, 2021; Tolar et al, 2020)." In the discussion, Page 10, "In the quest for AD therapies, emerging encouraging results suggest a clinically meaningful effect from immunotherapies aimed at clearing Ab-amyloid which recently also have led to the first FDA approval of a passive vaccine for treatment of AD (Demattos et al, 2012; Golde et al, 2009; Sevigny et al, 2016).
References should be updated with recent clinical studies about the efficacy of Aducanumab.
2. Schedule used to induce active immunization should be justified or referenced.
3. CADASIL is associated with high risk of stroke, dementia and migraine. Did the authors check the brain of TgN3R182C150 mice by MRI, for instance? This analysis could be interesting in order to increase the clinical impact and the translational value of the therapy.
4. It is indicated that the therapy did not affect to the vascular structure of the retina, suggesting that endogenous Notch signaling was not affected by vaccination and therefore the therapy is safe; however, additional tox analysis should be included to confirm the biocompatibility, such as blood pressure analysis, inflammation markers, renal and hepatic damage marker, ...

2. Significance:

Significance (Required)

The study is well written, very clear and the results are very promising. Some references related with the effect of Aducanumab in AD should ne updated. Safety and Tox analysis of the therapy should be complemented with additional assays.

3. How much time do you estimate the authors will need to complete the suggested revisions:

Estimated time to Complete Revisions (Required)

(Decision Recommendation)

Less than 1 month

4. Review Commons values the work of reviewers and encourages them to get credit for their work. Select 'Yes' below to register your reviewing activity at Publons; note that the content of your review will not be visible on Publons.

Reviewer Publons

Yes

Revision Plan

Manuscript number: RC-2022-01444
Corresponding author(s): Helena Karlström and Urban Lendahl

[The "revision plan" should delineate the revisions that authors intend to carry out in response to the points raised by the referees. It also provides the authors with the opportunity to explain their view of the paper and of the referee reports.

The document is important for the editors of affiliate journals when they make a first decision on the transferred manuscript. It will also be useful to readers of the reprint and help them to obtain a balanced view of the paper.

If you wish to submit a full revision, please use our "Full Revision" template. ***It is important to use the appropriate template to clearly inform the editors of your intentions.****]*

1. General Statements [optional]

We thank the reviewers for fair and constructive comments, and for good suggestions for how to further improve the manuscript. We also appreciate the comments on novelty and potential importance for future clinical approaches for CADASIL. Below we provide our point-by-point responses to the reviewers' comments. We start with a description of planned revisions, followed by describing changes already carried out in the transferred manuscript.

2. Description of the planned revisions

We plan to experimentally address the following points raised by the reviewers.

First, we will address the ASMA staining in the arterioles by isolating brain microvessels from the TgN3R182C150 mouse and WT mouse and strain for ASMA and perlecan/PDGFRb (stain pericytes and VSMC) to in more detail visualize that ASMA is only expressed in the arterioles and not in the pericytes in the capillaries (reviewer 1, point 4).

Second, we will assess the safety and toxicity of the active immunotherapy (reviewer 2, point 4). Specifically, we plan to analyse the structure and morphology of the kidneys from sham and vaccinated mice for renal damage by histology, H/E staining as well as creatinine levels in the serum. If the histology shows signs of damage (such as necrosis, apoptosis or granules in the tubules), we will stain the tissue with KIM1 for acute renal damage and caspase 3 for apoptosis. Moreover, we will analyse the inflammation C-Reactive Protein (CRP) marker in the serum by western blot analysis and/or ELISA and finally Neurofilament Light chain protein in the serum by SIMOA analysis to monitor if the vaccination is causing any neurodegeneration.

Third, we will improve the image quality for Figure 5A (reviewer 1, point 5).

Point-by-point responses to the reviewers' comments.

Revision Plan

Reviewer #1 (Evidence, reproducibility and clarity (Required)):

Summary:

This study have used immunogenic aggregates formed by recombinant Notch3 fragments EGF 1-5 that contain CADASIL NOTCH3 R133C and wild type NOTCH3 to inoculate a CADASIL mouse model TgN3R182C as an active immunisation therapy of CADASIL. The vaccinated mice showed a decreased deposition of NOTCH3 around brain capillaries, reduced blood NOTCH3 ECD and microglia activation, suggesting a potential novel therapy for future treatment of CADASIL. The lack of impact on retinal vasculature, body weight, and general behaviour of the treated mice indicate the safety of the therapy.

We thank the reviewer for the positive comments and for finding it to be a potential novel and safe therapy for CADASIL.

Major comments:

The results are interesting and the manuscript was carefully written and presented. However, the reduction of NOTCH3 in blood samples and the deposition around capillaries were so modest, plus there was no significant change of NOTCH3 deposition in arterioles. This questions the real effectiveness of this immunisation therapy when translating to clinical patients.

We thank the reviewers for the positive words on the manuscript and understand the concerns over the lack of effects in the arterioles. We still believe that observing NOTCH3 ECD reductions around capillaries and in the blood is a significant step forward and supports further endeavors in the area of active immunization, especially considering that the treatment is well tolerated with no overt NOTCH3-related toxicity effects. With this said, we of course realize that there would still be a long way towards clinical use, but all therapy developments have to start somewhere, and future studies (from us and others) can build on the data presented here. There are a number of possible explanations as to why there was not a significant reduction of NOTCH3 around the arterioles. It might be due to that the TgN3R182C150 model is a mild disease model which has a NOTCH3 accumulation onset around 6-7 months of age that starts in the capillaries and at later ages spreads to the arterioles, and that a different time axis or longer immunization period would also lead to a reduction of NOTCH3 in the arterioles, which can be addressed in future experiments. We however believe it was worthwhile to first explore an early immunization protocol, as it may pave the way to a treatment strategy that can be used early on the disease progress, maybe already at the pre-symptomatic phase.

Minor comments:

- Figure EV1 is redundant as Figure 2C has the same information.

We agree the figures are redundant and have deleted Figure EV1 in our new version and renamed EV2 and EV3 to EV1 and EV2, respectively.

- P5 last line "there was a prominent loss of monomeric NOTCH3 EGF1-5 when WT and R133C fragments were mixed as compared to incubating them separately (Fig 2C)". Why the aggregation is

more obvious when mutant protein fragment mixed with wild type comparing mutant fragment alone?

The reviewer raises an interesting point. While we do not strictly know why a mixing of mutated and wildtype fragments promote aggregation, our observations are in line with previous reports. Duering et al. Hum. Mol. Genet, 2011 used a single-particle approach SIFT (scanning for intensely fluorescent targets) to study the co-aggregation of WT and mutant NOTCH3 EGF1-5 proteins and reported that mixtures of WT and mutant proteins showed dual colour high-intensity signals, representing de novo aggregates. They also observed WT/R133C multimer formation over three days as compared to five days used in our study. It is also of note that most CADASIL cases are heterozygous for the NOTCH3 mutation, which suggests that wildtype and mutant NOTCH3 proteins co-exist in vivo, which also have been confirmed by co-immunoprecipitation experiments from cell lysates by Opherk et al. Hum. Mol. Genet. 2009. In the light of these previous reports, we performed a variety of ratio between WT and R133C NOTCH3 EGF1-5 proteins and opted for a 1:1 mixture since it showed the highest amount of multimers in relation to monomers after incubation.

- How was the dosage or concentration of aggregated protein (0.5 mg/ml) used for immunisation determined?

The dosage concentration was calculated by measuring the purified protein with a BCA absorbance assay. The protein was then mixed with the adjuvant or PBS to the desired concentration. This is now more clearly described in the Materials and Methods.

- P7 line 1-2, while using ASMA as a marker for arteries/arterioles, didn't the author see any expression of ASMA in pericytes (capillaries)?

We have used a direct fluorescent conjugated ASMA antibody for this mouse strain and another mouse strain with same mutation but higher NOTCH3 expression (described in Rutten et al, Acta Neuropathol Commun, 2015) as well as for the wildtype mice. In all cases, we find that pericytes have no or very low ASMA expression. This is in line with a report from Ghezali et al. Ann Neurol 2018, in which they used perlecan as a marker for pericytes in the rat Notch3 R169C mutation model, since ASMA did not stain the capillaries. The lack of ASMA staining in pericytes is in agreement with our previous large-scale single cell RNA-seq study of the mouse brain vasculature, where we find very little ASMA (ACTA2) gene expression in pericytes, while it is abundant in the vascular smooth muscle cells (Vanlandewijck et al., Nature 2018).
With this said, we will in the revised version include images on isolated brain microvessels from the N3TgR182C150 mouse model and WT that are stained with ASMA and perlecan/PDGFRb to clearly show that ASMA only stains the arterioles and not the capillaries, see Point 2 “Description of planned revisions” part above.

- In Figure 5A, the NOTCH3 ECD signal looks like similar between Shame and Vaccinated, although the quantifications seem significant in Figure 5B. The author may discuss the robustness of the quantitation method used and therefore the conclusion (i.e., active immunization with NOTCH3 EGF1-5 WT/R133C aggregates specifically reduces the amount of NOTCH3 aggregates around cerebral capillaries.).

We agree that Figure 5A is not clearly showing the differences between vaccinated and sham although highly significant. We have included more representative images and we have also improved the description of the quantification in the method part (page 18-19).

- In Figure 8B, what does the "% of microglia area" mean and how was it calculated?

The % of microglia area represents the percentage that the CD68 staining occupies per microglia area (stained by Iba1), meaning that the vaccinated mice contain more activated microglia compared to sham. This was calculated by measuring the area of the CD68 staining that colocalizes with the microglia. In the transferred version, we have improved the description of this in the text (page 18-19).

- Figure 9A doesn't seem to support the conclusion "There was also a trend towards more NOTCH3 ECD deposits inside or in close vicinity to microglia in vaccinated TgN3R182C150 mice compared to control or sham-vaccinated TgN3R182C150 mice, although the difference did not reach statistical significance", as it is not so convincing that the signals of the NOTCH3 ECD staining co-localise with microglia in the vaccinated sample. Besides, what's the meaning or functional significance about "% of microglia area", "number/1000 um2 microglia", and "average size per microglia"?

Thank you for the comments and we understand the concern, given the highly complex morphology and dynamics of microglia, which makes it difficult to describe potential differences in a straightforward manner. In the first graph in Figure 9B we show that the vaccinated mice had a higher percentage of microglia with NOTCH3 ECD deposits. However, these deposits did not occupy more area of the microglia (% microglia area, second graph), or showed a significant increase in the number of deposits per microglia area (number/1000 μm^2 microglia, third graph). The average size of these deposits did not increase in a significant way per microglia area (average size per microglia μm^2, fourth graph) in comparison to sham and non-vaccinated mice. We have rephrased the text to better indicate that the proportion of microglia with NOTCH3 deposits increased, while not the area or number of deposits within a microglial cell (Results, page 9).

Reviewer #1 (Significance (Required)):

CADASIL is the most common genetic small vessel disease that leads to cognitive defect and eventual vascular dementia. Current, there is no specific treatment available. Similar to a number of neurodegenerative conditions caused by protein accumulations like Alzheimer's disease, Parkinson disease and Huntington disease etc, NOTCH3 protein accumulation represents a key pathological change in CADASIL and therefore a drug target. The approach of active immunisation therapy described in this paper demonstrated a novel method for the treatment of this condition. Although the effectiveness of the therapy in the transgenic mouse model of CADASIL was yet highly impressive, this paper provides a proof-of-principle that the active immunisation is more or less functional and, most importantly, tolerable. One other advantage of this approach is that the immunisation therapy is not restricted to a specific NOTCH3 mutation. After further development this strategy could potentially benefit patients in the future.

This paper may be interested by researchers working on diseases that are caused by specific protein accumulation or aggregations.

My expertise is in the area of studying the molecular mechanisms of CADASIL and other genetic small vessel diseases.

Revision Plan

We thank the reviewer for these comments and that the manuscript provides proof-of-principle for a novel strategy towards a safe and tolerable therapy for future treatment of CADASIL patients. As such, we believe the study will be a useful platform and resource for future studies from us and others.

Reviewer #2 (Evidence, reproducibility and clarity (Required)):

The article submitted by Daniel V. Oliveira et al to Review Commons and titled "NOTCH3 active immunotherapy reduces NOTCH3 deposition in brain capillaries in a CADASIL mouse model" describes a novel active immunization therapy against the aggregated NOTCH3 mutant protein associated to CADASIL pathology. This strategy induces a significant reduction in NOTCH3 deposition around brain capillaries, increase of microglia activation and lowering of serum levels of NOTCH3, that demonstrates the potential clinical value of the therapy. In addition, the authors report that the therapy is safe and tolerable.
The study is well written, very clear and the results are very promising.

We thank the reviewer for positive comments on our work and pleased that you find the manuscript well and clearly written and containing promising data.

Same minor comments need to be clarified before publication.
1. In the introduction is indicated that ".....Aducanumab recently being approved for treatment of AD by the Food and Drug Association (Budd Haeberlein et al, 2017; Mintun et al, 2021; Tolar et al, 2020)." In the discussion, Page 10, "In the quest for AD therapies, emerging encouraging results suggest a clinically meaningful effect from immunotherapies aimed at clearing Ab-amyloid which recently also have led to the first FDA approval of a passive vaccine for treatment of AD (Demattos et al, 2012; Golde et al, 2009; Sevigny et al, 2016).
References should be updated with recent clinical studies about the efficacy of Aducanumab.

This is a valid point. We have updated the manuscript accordingly, and included a recent study on the effect of Aducanumab (Knopman et al. Alzheimers Dement. 2021).

2. Schedule used to induce active immunization should be justified or referenced.

We appreciate this comment and realize that we did not explain the rationale for the immunization scheme in the original version. We used a similar immunization schedule as used by Kontsekova et al. Alzheimer's Research & Therapy 2014 in their study of active immunization of a tau peptide in a transgenic rat Alzheimer disease model. This is now mentioned in the text on page 6. The slight modifications of the Kontsekova et al., scheme are also described in the Materials and Methods, page 15 (we made some slight modifications in order to be compatible to mouse and also immunized with an aggregated protein of 25kD instead of a 2 kD tau peptide).

3. CADASIL is associated with high risk of stroke, dementia and migraine. Did the authors check the brain of TgN3R182C150 mice by MRI, for instance? This analysis could be interesting in order to increase the clinical impact and the translational value of the therapy.

Thank you for pointing this out. We have previously reported MRI data on a more severe CADASIL mouse model (TgN3R182C350), which harbors the same transgene but expressed at a 2.3 times higher level compared to the mouse model used in this study (Rutten et al. Acta Neuropathol.

Commun. 2015 and Gravesteijn et al. Transl. Stroke Res. 2020). We did not observe any consistent differences on MRI or behavior between the TgN3R182C350 model and controls at 20 months of age. From this data we do not expect to see any differences on MRI in our milder model at 7 months of age. We have however included a short description of the previous MRI data in the transferred version on page 12.

4. It is indicated that the therapy did not affect to the vascular structure of the retina, suggesting that endogenous Notch signaling was not affected by vaccination and therefore the therapy is safe; however, additional tox analysis should be included to confirm the biocompatibility, such as blood pressure analysis, inflammation markers, renal and hepatic damage marker.

Thank you for pointing out this important issue regarding the safety aspects for our active immunotherapy. We will address this by additional experiments of the renal damage by histology and inflammation markers and neuronal damage markers in the serum with western blotting/ELISA and SIMOA, which we have written in more detail in Point 2 “Description of planned revisions” part above.

The suggestions for blood pressure and hepatic damage analysis are valid but would require that we restart novel series of experiments from scratch, as we currently do not have immunized mice up and running. Such an experiment would take several months, and with the subsequent analysis, most likely more than a year. We believe this time axis is too long, and we therefore respectfully suggest waiving these experiments. Also, we unfortunately did not collect livers from the first round of animals, so analysis of livers would have a similarly long time axis.

Reviewer #2 (Significance (Required)):

The study is well written, very clear and the results are very promising. Some references related with the effect of Aducanumab in AD should ne updated. Safety and Tox analysis of the therapy should be complemented with additional assays.

We thank the reviewer for these positive comments. We have updated the manuscript with a new reference regarding the effect of Aducanumab. For the safety and tox analysis we will do additional experimental analysis which we have addressed above in “Description of planned revisions”.

3. Description of the revisions that have already been incorporated in the transferred manuscript

Please insert a point-by-point reply describing the revisions that were already carried out and included in the transferred manuscript. If no revisions have been carried out yet, please leave this section empty.

We deleted Figure EV1 in our new version and renamed EV2 and EV3 to EV1 and EV2, respectively. (Reviewer 1, point 1).

We provide an improved description of how the dosage concentration was calculated by measuring the purified protein with a BCA absorbance assay (Reviewer 1, point 3) (Materials and Methods, page 15).

We have improved the description of the quantification of the immunohistochemistry in the method part (Reviewer 1, point 5) (Materials and Methods, page 18-19).

We have improved the description of microglia in the quantification in the method part (Reviewer 1, point 6 and 7) (Materials and Methods, page 18-19).

We have rephrased the text to better indicate that the proportion of microglia with NOTCH3 deposits increased, while not the area or number of deposits within a microglial cell (Results, page 9).

We have updated the manuscript accordingly, and included a recent study on the effect of Aducanumab (Knopman et al. Alzheimers Dement. 2021). (Reviewer 2, point 1).

We have provided a rationale and a more detailed description of the immunization protocol. (Reviewer 2, point 2) (Materials and Methods, page 15).

We have included a short description of the previous MRI data in the transferred version. (Reviewer 2, point 3), (Discussion, page 12)

We have included the following new references (Vanlandewijck et al., Nature 2018, Knopman et al. Alzheimers Dement. 2021, Kontsekova et al. Alzheimer's Research & Therapy 2014, Gravesteijn et al. Transl. Stroke Res. 2020).

4. Description of analyses that authors prefer not to carry out

Please include a point-by-point response explaining why some of the requested data or additional analyses might not be necessary or cannot be provided within the scope of a revision. This can be due to time or resource limitations or in case of disagreement about the necessity of such additional data given the scope of the study. Please leave empty if not applicable.

The suggestions for blood pressure and hepatic damage analysis are valid but would require that we restart novel series of experiments from scratch, as we currently do not have immunized mice up and running. Such an experiment would take several months, and with the subsequent analysis, most likely more than a year. We believe this time axis is too long, and we therefore respectfully suggest waiving these experiments. Also, we unfortunately did not collect livers from the first round of animals, so analysis of livers would have a similar time axis.

12th Jul 2022

Dear Dr. Karlstrom,

Thank you for the submission of your manuscript to our editorial offices. I have now had the opportunity to read it, together with the referees' reports and your rebuttal letter, and to discuss them with the other members of our editorial team.

We agree that the study fits the scope of the journal, and we appreciate that you are willing to address most of the points raised by the reviewers. We thus encourage you to submit a revised version of your manuscript, including the modifications and revisions described in your point-by-point letter. Acceptance of the manuscript will entail a second round of review. EMBO Molecular Medicine encourages a single round of revision only and therefore, acceptance or rejection of the manuscript will depend on the completeness of your responses included in the next, final version of the manuscript. For this reason, and to save you from any frustrations in the end, I would strongly advise against returning an incomplete revision.

***

When submitting your revised manuscript, please carefully review the instructions that follow below. Failure to include requested items will delay the evaluation of your revision:

We require:

1) A .docx formatted version of the manuscript text (including legends for main figures, EV figures and tables). Please make sure that the changes are highlighted to be clearly visible.

2) Individual production quality figure files as .eps, .tif, .jpg (one file per figure). For guidance, download the 'Figure Guide PDF' (https://www.embopress.org/page/journal/17574684/authorguide#figureformat).

3) A .docx formatted letter INCLUDING the reviewers' reports and your detailed point-by-point responses to their comments. As part of the EMBO Press transparent editorial process, the point-by-point response is part of the Review Process File (RPF), which will be published alongside your paper.

4) A complete author checklist, which you can download from our author guidelines (https://www.embopress.org/page/journal/17574684/authorguide#submissionofrevisions). Please insert information in the checklist that is also reflected in the manuscript. The completed author checklist will also be part of the RPF.

5) It is mandatory to include a 'Data Availability' section after the Materials and Methods. Before submitting your revision, primary datasets produced in this study need to be deposited in an appropriate public database, and the accession numbers and database listed under 'Data Availability'. Please remember to provide a reviewer password if the datasets are not yet public (see https://www.embopress.org/page/journal/17574684/authorguide#dataavailability).
In case you have no data that requires deposition in a public database, please state so in this section. Note that the Data Availability Section is restricted to new primary data that are part of this study.

6) For data quantification: please specify the name of the statistical test used to generate error bars and P values, the number

(n) of independent experiments (specify technical or biological replicates) underlying each data point and the test used to calculate p-values in each figure legend. The figure legends should contain a basic description of n, P and the test applied. Graphs must include a description of the bars and the error bars (s.d., s.e.m.).

7) We would also encourage you to include the source data for figure panels that show essential data. Numerical data should be provided as individual .xls or .csv files (including a tab describing the data). For blots or microscopy, uncropped images should be submitted (using a zip archive if multiple images need to be supplied for one panel). Additional information on source data

and instruction on how to label the files are available at
<https://www.embopress.org/page/journal/17574684/authorguide#sourcedata>.

8) Our journal encourages inclusion of *data citations in the reference list* to directly cite datasets that were re-used and obtained from public databases. Data citations in the article text are distinct from normal bibliographical citations and should directly link to the database records from which the data can be accessed. In the main text, data citations are formatted as follows: "Data ref: Smith et al, 2001" or "Data ref: NCBI Sequence Read Archive PRJNA342805, 2017". In the Reference list, data citations must be labeled with "[DATASET]". A data reference must provide the database name, accession number/identifiers and a resolvable link to the landing page from which the data can be accessed at the end of the reference. Further instructions are available at <https://www.embopress.org/page/journal/17574684/authorguide#referencesformat>.

9) We replaced Supplementary Information with Expanded View (EV) Figures and Tables that are collapsible/expandable online. A maximum of 5 EV Figures can be typeset. EV Figures should be cited as 'Figure EV1, Figure EV2" etc... in the text and their respective legends should be included in the main text after the legends of regular figures.
- For the figures that you do NOT wish to display as Expanded View figures, they should be bundled together with their legends in a single PDF file called *Appendix*, which should start with a short Table of Content. Appendix figures should be referred to in the main text as: "Appendix Figure S1, Appendix Figure S2" etc.
- Additional Tables/Datasets should be labeled and referred to as Table EV1, Dataset EV1, etc. Legends have to be provided in a separate tab in case of .xls files. Alternatively, the legend can be supplied as a separate text file (README) and zipped together with the Table/Dataset file.
See detailed instructions here:
<https://www.embopress.org/page/journal/17574684/authorguide#expandedview>.

10) The paper explained: EMBO Molecular Medicine articles are accompanied by a summary of the articles to emphasize the major findings in the paper and their medical implications for the non-specialist reader. Please provide a draft summary of your article highlighting
- the medical issue you are addressing,
- the results obtained and
- their clinical impact.
This may be edited to ensure that readers understand the significance and context of the research. Please refer to any of our published articles for an example.

11) For more information: There is space at the end of each article to list relevant web links for further consultation by our readers. Could you identify some relevant ones and provide such information as well? Some examples are patient associations, relevant databases, OMIM/proteins/genes links, author's websites, etc...

12) Author contributions: the contribution of every author must be detailed in a separate section (before the acknowledgments).

13) Conflict of interest: We updated our journal's competing interests policy in January 2022 and request authors to consider both actual and perceived competing interests. Please review the policy https://www.embopress.org/competing-interests and update your competing interests if necessary.

14) Every published paper now includes a 'Synopsis' to further enhance discoverability. Synopses are displayed on the journal webpage and are freely accessible to all readers. They include a short stand first (maximum of 300 characters, including space) as well as 2-5 one-sentences bullet points that summarizes the paper. Please write the bullet points to summarize the key NEW findings. They should be designed to be complementary to the abstract - i.e. not repeat the same text. We encourage inclusion of key acronyms and quantitative information (maximum of 30 words / bullet point). Please use the passive voice. Please attach these in a separate file or send them by email, we will incorporate them accordingly.
Please also suggest a striking image or visual abstract to illustrate your article as a PNG file 550 px wide x 300-600 px high.

15) As part of the EMBO Publications transparent editorial process initiative (see our Editorial at http://embomolmed.embopress.org/content/2/9/329), EMBO Molecular Medicine will publish online a Review Process File (RPF) to accompany accepted manuscripts.
In the event of acceptance, this file will be published in conjunction with your paper and will include the anonymous referee reports, your point-by-point response and all pertinent correspondence relating to the manuscript. Let us know whether you agree with the publication of the RPF and as here, if you want to remove or not any figures from it prior to publication.
Please note that the Authors checklist will be published at the end of the RPF.

EMBO Molecular Medicine has a "scooping protection" policy, whereby similar findings that are published by others during review or revision are not a criterion for rejection. Should you decide to submit a revised version, I do ask that you get in touch after three months if you have not completed it, to update us on the status.

I look forward to receiving your revised manuscript.

Yours sincerely,

Lise Roth

Lise Roth, PhD
Senior Editor
EMBO Molecular Medicine

Manuscript number: EMM-2022-16556-V2
Corresponding author(s): Helena Karlström and Urban Lendahl

We thank the reviewers for fair and constructive comments, and for good suggestions for how to further improve the manuscript. We also appreciate the comments on novelty and potential importance for future clinical approaches for CADASIL. Below we provide our point-by-point responses to the reviewers' comments.

Point-by-point responses to the reviewers' comments.

Reviewer #1 (Evidence, reproducibility and clarity (Required)):

Summary:

This study have used immunogenic aggregates formed by recombinant Notch3 fragments EGF 1-5 that contain CADASIL NOTCH3 R133C and wild type NOTCH3 to inoculate a CADASIL mouse model TgN3R182C as an active immunisation therapy of CADASIL. The vaccinated mice showed a decreased deposition of NOTCH3 around brain capillaries, reduced blood NOTCH3 ECD and microglia activation, suggesting a potential novel therapy for future treatment of CADASIL. The lack of impact on retinal vasculature, body weight, and general behaviour of the treated mice indicate the safety of the therapy.

We thank the reviewer for the positive comments and for finding it to be a potential novel and safe therapy for CADASIL.

Major comments:

The results are interesting and the manuscript was carefully written and presented. However, the reduction of NOTCH3 in blood samples and the deposition around capillaries were so modest, plus there was no significant change of NOTCH3 deposition in arterioles. This questions the real effectiveness of this immunisation therapy when translating to clinical patients.

We appreciate the positive words on the manuscript and understand the concerns over the lack of effects in the arterioles. We still believe that observing NOTCH3 ECD reductions around capillaries and in the blood is a significant step forward and supports further endeavors in the area of active immunization, especially considering that the treatment is well tolerated with no overt NOTCH3-related toxicity effects. With this said, we of course realize that there would still be a long way towards clinical use, but all therapy developments have to start somewhere, and future studies (from us and others) can build on the data presented here. There are a number of possible explanations as to why there was not a significant reduction of NOTCH3 around the arterioles. It might be due to that the TgN3R182C150 model is a mild disease model which has a NOTCH3 accumulation onset around 6-7 months of age that starts in the capillaries and at later ages spreads to the arterioles, and that a different time axis or longer immunization period would also provide a reduction of NOTCH3 in the arterioles, which can be addressed in future experiments. We however believe it was worthwhile to first explore an early immunization protocol, as it may pave the way to a treatment strategy that can be used early on the disease progress, maybe already at the pre-symptomatic phase.

Minor comments:

- Figure EV1 is redundant as Figure 2C has the same information.

We agree the figures are redundant and have deleted Figure EV1 in our new version. The EV1 figure is substituted with a new figure that shows staining of alpha smooth muscle actin (ASMA) in arteries/arterioles and not capillaries in isolated mouse brain vessels, in response to Reviewer 1's comment in point 4 below.

- P5 last line "there was a prominent loss of monomeric NOTCH3 EGF1-5 when WT and R133C fragments were mixed as compared to incubating them separately (Fig 2C)". Why the aggregation is more obvious when mutant protein fragment mixed with wild type comparing mutant fragment alone?

The reviewer raises an interesting point. While we do not strictly know why a mixing of mutated and wildtype fragments promotes aggregation, our observations are in line with previous reports. Duering et al. Hum. Mol. Genet, 2011 used a single-particle approach SIFT (scanning for intensely fluorescent targets) to study the co-aggregation of WT and mutant NOTCH3 EGF1-5 proteins and reported that mixtures of WT and mutant proteins showed dual colour high-intensity signals, representing de novo aggregates. They also observed WT/R133C multimer formation over three days as compared to five days used in our study. It is also of note that most CADASIL cases are heterozygous for the NOTCH3 mutation, which suggests that wildtype and mutant NOTCH3 proteins co-exist in vivo, which also has been confirmed by co-immunoprecipitation experiments from cell lysates by Opherk et al. Hum. Mol. Genet. 2009. In the light of these previous reports, we explored a range of ratios between WT and R133C NOTCH3 EGF1-5 proteins and opted for a 1:1 mixture since it showed the highest amount of multimers in relation to monomers after incubation.

- How was the dosage or concentration of aggregated protein (0.5 mg/ml) used for immunisation determined?

The dosage concentration was calculated by measuring the purified protein with a BCA absorbance assay. The protein was then mixed with the adjuvant or PBS to the desired concentration. This is now more clearly described in the Materials and Methods (page 17).

- P7 line 1-2, while using ASMA as a marker for arteries/arterioles, didn't the author see any expression of ASMA in pericytes (capillaries)?

We have used a direct fluorescent conjugated ASMA antibody for this mouse strain and another mouse strain with the same mutation but higher NOTCH3 expression (described in Rutten et al, Acta Neuropathol Commun, 2015) as well as for the wildtype (WT) mice. In all cases, we find that pericytes have no or very low ASMA expression. This is in line with a report from Ghezali et al. Ann Neurol 2018, in which they used perlecan as a marker for pericytes in the rat Notch3 R169C mutation model, since ASMA did not stain the capillaries. The lack of ASMA staining in pericytes is in agreement with our previous large-scale single cell RNA-seq study of the mouse brain vasculature, where we find very little ASMA (ACTA2) gene expression in pericytes, while it is abundant in the vascular smooth muscle cells (Vanlandewijck et al., Nature 2018).

With this said, we have in this version included images on isolated brain microvessels from the N3TgR182C150 mouse model and WT that are stained with ASMA and perlecan/PDGFRβ/NG2 that clearly show that ASMA only stains the arterioles and not the capillaries (PDGFRβ and NG2 stain both VSMC and pericytes and perlecan stain VSMC, pericytes and endothelial cells) (new Figure EV1).

- In Figure 5A, the NOTCH3 ECD signal looks like similar between Shame and Vaccinated, although the quantifications seem significant in Figure 5B. The author may discuss the robustness of the quantitation method used and therefore the conclusion (i.e., active immunization with NOTCH3 EGF1-5 WT/R133C aggregates specifically reduces the amount of NOTCH3 aggregates around cerebral capillaries.).

We agree that Figure 5A is not clearly showing the differences between vaccinated and sham although highly significant. We have included more representative images and we have also improved the description of the quantification in the method part (page 20-21).

- In Figure 8B, what does the "% of microglia area" mean and how was it calculated?

The % of microglia area represents the percentage that the CD68 staining occupies per microglia area (stained by Iba1), meaning that the vaccinated mice contain more activated microglia compared to sham. This was calculated by measuring the area of the CD68 staining that colocalizes with the microglia. In the revised version, we have improved the description of this in the text (page 20-21).

- Figure 9A doesn't seem to support the conclusion "There was also a trend towards more NOTCH3 ECD deposits inside or in close vicinity to microglia in vaccinated TgN3R182C150 mice compared to control or sham-vaccinated TgN3R182C150 mice, although the difference did not reach statistical significance", as it is not so convincing that the signals of the NOTCH3 ECD staining co-localise with microglia in the vaccinated sample. Besides, what's the meaning or functional significance about "% of microglia area", "number/1000 um2 microglia", and "average size per microglia"?

Thank you for the comments and we understand the concern, given the highly complex morphology and dynamics of microglia, which makes it difficult to describe potential differences in a straightforward manner. In the first graph in Figure 7C we show that the vaccinated mice had a higher percentage of microglia with NOTCH3 ECD deposits. However, these deposits did not occupy more area of the microglia (% microglia area, second graph), or showed a significant increase in the number of deposits per microglia area (number/1000 μm^2 microglia, third graph). The average size of these deposits did not increase in a significant way per microglia area (average size per microglia μm^2, fourth graph) in comparison to sham and non-vaccinated mice. We have rephrased the text to better indicate that the proportion of microglia with NOTCH3 deposits increased, while the area or number of deposits within a microglial cell did not (Results, page 10).

Reviewer #1 (Significance (Required)):

CADASIL is the most common genetic small vessel disease that leads to cognitive defect and eventual vascular dementia. Current, there is no specific treatment available. Similar to a number of neurodegenerative conditions caused by protein accumulations like Alzheimer's disease, Parkinson disease and Huntington disease etc, NOTCH3 protein accumulation represents a key pathological change in CADASIL and therefore a drug target. The approach of active immunisation therapy described in this paper demonstrated a novel method for the treatment of this condition. Although the effectiveness of the therapy in the transgenic mouse model of CADASIL was yet highly impressive, this paper provides a proof-of-principle that the active immunisation is more or less functional and, most importantly, tolerable. One other advantage of this approach is that the immunisation therapy is not restricted to a specific NOTCH3 mutation. After further development this strategy could potentially benefit patients in the future.

This paper may be interested by researchers working on diseases that are caused by specific protein accumulation or aggregations.

My expertise is in the area of studying the molecular mechanisms of CADASIL and other genetic small vessel diseases.

We thank the reviewer for these comments and that the manuscript provides proof-of-principle for a novel strategy towards a safe and tolerable therapy for future treatment of CADASIL

patients. As such, we believe the study will be a useful platform and resource for future studies from us and others.

Reviewer #2 (Evidence, reproducibility and clarity (Required)):

The article submitted by Daniel V. Oliveira et al to Review Commons and titled "NOTCH3 active immunotherapy reduces NOTCH3 deposition in brain capillaries in a CADASIL mouse model" describes a novel active immunization therapy against the aggregated NOTCH3 mutant protein associated to CADASIL pathology. This strategy induces a significant reduction in NOTCH3 deposition around brain capillaries, increase of microglia activation and lowering of serum levels of NOTCH3, that demonstrates the potential clinical value of the therapy. In addition, the authors report that the therapy is safe and tolerable.
The study is well written, very clear and the results are very promising.

We thank the reviewer for positive comments on our work and are pleased that you find the manuscript well and clearly written and containing promising data.

Same minor comments need to be clarified before publication.
1. In the introduction is indicated that ".....Aducanumab recently being approved for treatment of AD by the Food and Drug Association (Budd Haeberlein et al, 2017; Mintun et al, 2021; Tolar et al, 2020)." In the discussion, Page 10, "In the quest for AD therapies, emerging encouraging results suggest a clinically meaningful effect from immunotherapies aimed at clearing Ab-amyloid which recently also have led to the first FDA approval of a passive vaccine for treatment of AD (Demattos et al, 2012; Golde et al, 2009; Sevigny et al, 2016).
References should be updated with recent clinical studies about the efficacy of Aducanumab.

This is a valid point. We have updated the manuscript accordingly and included a recent study on the effect of Aducanumab (Knopman et al. Alzheimers Dement. 2021) (page 5 and 12).

2. Schedule used to induce active immunization should be justified or referenced.

We appreciate this comment and realize that we did not explain the rationale for the immunization scheme in the original version. We used a similar immunization schedule as previously deployed by Kontsekova et al. Alzheimer's Research & Therapy 2014 in their study of active immunization of a tau peptide in a transgenic rat Alzheimer disease model. This is now mentioned in the text on page 7. The slight modifications of the Kontsekova et al., scheme are also described in the Materials and Methods, page 17 (we made some slight modifications in order to be compatible to mouse and also immunized with an aggregated protein of 25 kD instead of a 2 kD tau peptide).

3. CADASIL is associated with high risk of stroke, dementia and migraine. Did the authors check the brain of TgN3R182C150 mice by MRI, for instance? This analysis could be interesting in order to increase the clinical impact and the translational value of the therapy.

Thank you for pointing this out. We have previously reported MRI data on a more severe CADASIL mouse model (TgN3R182C350), which harbors the same transgene but expressed at a 2.3 times higher level compared to the mouse model used in this study (Rutten et al. Acta Neuropathol. Commun. 2015 and Gravesteijn et al. Transl. Stroke Res. 2020). We did not observe any consistent differences on MRI or behavior between the TgN3R182C350 model and controls at 20 months of age. From these data we do not expect to see any differences on MRI in our milder model at 7 months of age. We have however included a short description of the previous MRI data in the revised version on page 14.

4. It is indicated that the therapy did not affect to the vascular structure of the retina, suggesting

that endogenous Notch signaling was not affected by vaccination and therefore the therapy is safe; however, additional tox analysis should be included to confirm the biocompatibility, such as blood pressure analysis, inflammation markers, renal and hepatic damage marker.

Thank you for pointing out the important issue regarding the safety aspects for our active immunotherapy. We have addressed this by additional experiments of the renal damage by H&E histology and staining for the caspase 3 apoptosis marker as well as inflammation marker C-Reactive Protein (CRP) and neuronal damage marker Neurofilament Light chain in the serum with ELISA and Simoa, respectively. We have included a new paragraph in the Result section on page 11 and a description in Material and Methods, page 21-23 and a new EV3 figure, which shows histology and immunohistochemistry staining of the kidney and CRP/ Neurofilament Light chain concentration in the serum of sham and vaccinated mice.

The suggestions for blood pressure and hepatic damage analysis are valid but would require that we restart a novel series of experiments from scratch, as we currently do not have immunized mice up and running. Such an experiment would take several months, and with the subsequent analysis, most likely more than a year. We believe this time axis is too long, and we therefore respectfully suggest waiving these experiments. Also, we unfortunately did not collect livers from the first round of animals, so analysis of livers would have a similarly long time axis.

Reviewer #2 (Significance (Required)):

The study is well written, very clear and the results are very promising. Some references related with the effect of Aducanumab in AD should ne updated. Safety and Tox analysis of the therapy should be complemented with additional assays.

We thank the reviewer for these positive comments. We have updated the manuscript with a new reference regarding the effect of Aducanumab. For the safety and tox analysis we have addressed this by including new experimental analysis on kidney and serum.

We have also included the following new references (Vanlandewijck et al., Nature 2018, Knopman et al. Alzheimers Dement. 2021, Kontsekova et al. Alzheimer's Research & Therapy 2014, Gravesteijn et al. Transl. Stroke Res. 2020, Boulay AC et al. J Vis Exp. 2015 and Rodig et al. Cold Spring Harb Protoc 2021).

25th Oct 2022

Dear Dr. Karlstrom,

Thank you for the submission of your manuscript to EMBO Molecular Medicine. We have now received the reports from the referees who re-reviewed your manuscript. As you will see below, they are now supportive of publication, and I am therefore pleased to inform you that we will be able to accept your manuscript once the following editorial points will be addressed:

1/ Manuscript text:
- Please address the queries from our data editors in the Data edited MS file in track changes mode. Remove the text in blue, and only keep in track changes mode any new modification.
- Kindly suggest up to 5 keywords.
- Remove "data not shown". As per our guidelines on "Unpublished Data", the journal does not permit citation of "Data not shown". All data referred to in the paper should be displayed in the main or Expanded View figures.
- Material and methods:
o Please provide the origin of the mice, as well as gender and age at time of experiments.
o Please provide the commercial origin of the cells, and if they were tested for mycoplasma contamination.
- The figure legends should be placed after the references.
- Author contributions: CRediT has replaced the traditional author contributions section because it offers a systematic machine-readable author contributions format that allows for more effective research assessment. Please remove the Authors Contributions from the manuscript and use the free text boxes beneath each contributing author's name in our system to add specific details on the author's contribution. More information is available in our guide to authors.
- Conflict of interest: please update the title of this section to "Disclosure statement and competing interests".

2/ Figures:
- Please provide exact p values (including for ns, non significant) in the figures or their legends. Some people found that to keep the figures clear, providing a supplemental table in the Appendix with all exact p-values was preferable. You are welcome to do this if you want to.
- Figures need to fit onto 1 page, please adjust accordingly (your figure 7 currently spans two pages).
- There are 6 datasets linked to figures: if these are Source Data, please rename them and zip your files to have 1 Source Data file per figure. If they are not, they need to be renamed Datasets EV1-6 and legends and callouts should be added in the manuscript file.

3/ Thank you for providing The Paper Explained. Please include the text in the manuscript file.

4/ Thank you for providing a nice synopsis image. Please upload it as a png/jpeg/tiff file 550 px wide x 300-600 px high, and make sure that the text remains legible.

5/ As part of the EMBO Publications transparent editorial process initiative (see our Editorial at http://embomolmed.embopress.org/content/2/9/329), EMBO Molecular Medicine will publish online a Review Process File (RPF) to accompany accepted manuscripts.
This file will be published in conjunction with your paper and will include the anonymous referee reports, your point-by-point response and all pertinent correspondence relating to the manuscript. Let us know whether you agree with the publication of the RPF and as here, if you want to remove or not any figures from it prior to publication.
Please note that the Authors checklist will be published at the end of the RPF.

I look forward to receiving your revised manuscript.

Yours sincerely,

Lise Roth

Lise Roth, PhD
Senior Editor
EMBO Molecular Medicine

***** Reviewer's comments *****

Referee #1 (Comments on Novelty/Model System for Author):

The approach of active immunisation therapy described in this paper demonstrated a novel method for the treatment of CADASIL. It provides a proof-of-principle that the active immunisation is more or less functional and, most importantly, tolerable. One other advantage of this approach is that the immunisation therapy is not restricted to a specific NOTCH3 mutation. After further development this strategy could potentially benefit patients in the future.

Referee #1 (Remarks for Author):

The author has adequately addressed my comments on the original version and made corresponding changes in the new version. I have no further comments and agree to publish the paper.

Referee #2 (Comments on Novelty/Model System for Author):

This is an interesting and novel article focused in a new approach for CADASIL. The authors have successfully responded and included all comments indicated in the first review. I fully recommend the article for publication in EMBO journal.

***

Rev_Com_number: RC-2022-01444
New_manu_number: EMM-2022-16556-V2
Corr_author: Karlstrom
Title: Active immunotherapy reduces NOTCH3 deposition in brain capillaries in a CADASIL mouse model

The authors addressed the minor editorial issues.

28th Nov 2022

Dear Dr. Karlstrom,

Thank you for providing the corrected manuscript. I am pleased to inform you that your manuscript is accepted for publication and is now being sent to our publisher to be included in the next available issue of EMBO Molecular Medicine.

We would like to remind you that as part of the EMBO Publications transparent editorial process initiative, EMBO Molecular Medicine will publish a Review Process File online to accompany accepted manuscripts. If you do NOT want the file to be published or would like to exclude figures, please immediately inform the editorial office via e-mail.

Please read below for additional IMPORTANT information regarding your article, its publication and the production process.

Congratulations on your interesting work!

With kind regards,

Lise Roth

Lise Roth, Ph.D
Senior Editor
EMBO Molecular Medicine

Follow us on Twitter @EmboMolMed

EMBO Press Author Checklist

Corresponding Author Name: Helena Karlström and Urban Lendahl
Journal Submitted to: EMBO Molecular Medicine
Manuscript Number: EMM-2022-16556-V2

USEFUL LINKS FOR COMPLETING THIS FORM
The EMBO Journal - Author Guidelines
EMBO Reports - Author Guidelines
Molecular Systems Biology - Author Guidelines
EMBO Molecular Medicine - Author Guidelines

Reporting Checklist for Life Science Articles (updated January 2022)
This checklist is adapted from Materials Design Analysis Reporting (MDAR) Checklist for Authors. MDAR establishes a minimum set of requirements in transparent reporting in the life sciences (see Statement of Task: 10.31222/osf.io/9sm4x). Please follow the journal's guidelines in preparing your manuscript.
Please note that a copy of this checklist will be published alongside your article.

Abridged guidelines for figures
1. Data
The data shown in figures should satisfy the following conditions:
- the data were obtained and processed according to the field's best practice and are presented to reflect the results of the experiments in an accurate and unbiased manner.
- ideally, figure panels should include only measurements that are directly comparable to each other and obtained with the same assay.
- plots include clearly labeled error bars for independent experiments and sample sizes. Unless justified, error bars should not be shown for technical replicates.
- if n<5, the individual data points from each experiment should be plotted. Any statistical test employed should be justified.
- Source Data should be included to report the data underlying figures according to the guidelines set out in the authorship guidelines on Data Presentation.

2. Captions
Each figure caption should contain the following information, for each panel where they are relevant:
- a specification of the experimental system investigated (eg cell line, species name).
- the assay(s) and method(s) used to carry out the reported observations and measurements.
- an explicit mention of the biological and chemical entity(ies) that are being measured.
- an explicit mention of the biological and chemical entity(ies) that are altered/varied/perturbed in a controlled manner.
- the exact sample size (n) for each experimental group/condition, given as a number, not a range;
- a description of the sample collection allowing the reader to understand whether the samples represent technical or biological replicates (including how many animals, litters, cultures, etc.).
- a statement of how many times the experiment shown was independently replicated in the laboratory.
- definitions of statistical methods and measures:
  - common tests, such as t-test (please specify whether paired vs. unpaired), simple χ2 tests, Wilcoxon and Mann-Whitney tests, can be unambiguously identified by name only, but more complex techniques should be described in the methods section;
  - are tests one-sided or two-sided?
  - are there adjustments for multiple comparisons?
  - exact statistical test results, e.g., P values = x but not P values < x;
  - definition of 'center values' as median or average;
  - definition of error bars as s.d. or s.e.m.

Please complete ALL of the questions below.
Select "Not Applicable" only when the requested information is not relevant for your study.

Materials

**Newly Created Materials**	**Information included in the manuscript?**	**In which section is the information available?** (Reagents and Tools Table, Materials and Methods, Figures, Data Availability Section)
New materials and reagents need to be available; do any restrictions apply?	Not Applicable	

**Antibodies**	**Information included in the manuscript?**	**In which section is the information available?** (Reagents and Tools Table, Materials and Methods, Figures, Data Availability Section)
For **antibodies** provide the following information:<br>- Commercial antibodies: RRID (if possible) or supplier name, catalogue number and or/clone number<br>- Non-commercial: RRID or citation	Yes	Materials and Methods

**DNA and RNA sequences**	**Information included in the manuscript?**	**In which section is the information available?** (Reagents and Tools Table, Materials and Methods, Figures, Data Availability Section)
**Short novel DNA or RNA including primers, probes**: provide the sequences.	Yes	Materials and Methods

**Cell materials**	**Information included in the manuscript?**	**In which section is the information available?** (Reagents and Tools Table, Materials and Methods, Figures, Data Availability Section)
**Cell lines:** Provide species information, strain. Provide accession number in repository **OR** supplier name, catalog number, clone number, and/**OR** RRID.	Yes	Materials and Methods
**Primary cultures:** Provide species, strain, sex of origin, genetic modification status.	Not Applicable	
Report if the cell lines were recently **authenticated** (e.g., by STR profiling) and tested for mycoplasma contamination.	Not Applicable	

**Experimental animals**	**Information included in the manuscript?**	**In which section is the information available?** (Reagents and Tools Table, Materials and Methods, Figures, Data Availability Section)
**Laboratory animals or Model organisms:** Provide species, strain, sex, age, genetic modification status. Provide accession number in repository **OR** supplier name, catalog number, clone number, **OR** RRID.	Yes	Material and Methods and references
**Animal observed in or captured from the field:** Provide species, sex, and age where possible.	Not Applicable	
Please detail **housing and husbandry conditions**.	Yes	Matrial and Methods and Ethical permit number

**Plants and microbes**	**Information included in the manuscript?**	**In which section is the information available?** (Reagents and Tools Table, Materials and Methods, Figures, Data Availability Section)
**Plants:** provide species and strain, ecotype and cultivar where relevant, unique accession number if available, and source (including location for collected wild specimens).	Not Applicable	
**Microbes:** provide species and strain, unique accession number if available, and source.	Not Applicable	

**Human research participants**	**Information included in the manuscript?**	**In which section is the information available?** (Reagents and Tools Table, Materials and Methods, Figures, Data Availability Section)
If collected and within the bounds of privacy constraints report on age, sex and gender or ethnicity for all study participants.	Not Applicable	

**Core facilities**	**Information included in the manuscript?**	**In which section is the information available?** (Reagents and Tools Table, Materials and Methods, Figures, Data Availability Section)
If your work benefited from core facilities, was their service mentioned in the acknowledgments section?	Not Applicable	

Design

Study protocol	Information included in the manuscript?	In which section is the information available? (Reagents and Tools Table, Materials and Methods, Figures, Data Availability Section)
If study protocol has been **pre-registered, provide DOI in the manuscript**. For clinical trials, provide the trial registration number **OR** cite DOI.	Not Applicable	
Report the **clinical trial registration number** (at ClinicalTrials.gov or equivalent), where applicable.	Not Applicable	

Laboratory protocol	Information included in the manuscript?	In which section is the information available? (Reagents and Tools Table, Materials and Methods, Figures, Data Availability Section)
Provide DOI OR other citation details if **external detailed step-by-step protocols** are available.	Not Applicable	

Experimental study design and statistics	Information included in the manuscript?	In which section is the information available? (Reagents and Tools Table, Materials and Methods, Figures, Data Availability Section)
Include a statement about **sample size** estimate even if no statistical methods were used.	Yes	Materials and Methods, Figure legends
Were any steps taken to minimize the effects of subjective bias when allocating animals/samples to treatment (e.g. **randomization procedure**)? If yes, have they been described?	Yes	Materials and Methods
Include a statement about **blinding** even if no blinding was done.	Not Applicable	
Describe **inclusion/exclusion criteria** if samples or animals were excluded from the analysis. Were the criteria pre-established?<br><br>If sample or data points were omitted from analysis, report if this was due to attrition or intentional exclusion and provide justification.	Not Applicable	
For every figure, are **statistical tests** justified as appropriate? Do the data meet the assumptions of the tests (e.g., normal distribution)? Describe any methods used to assess it. Is there an estimate of variation within each group of data? Is the variance similar between the groups that are being statistically compared?	Yes	Materials and Methods

Sample definition and in-laboratory replication	Information included in the manuscript?	In which section is the information available? (Reagents and Tools Table, Materials and Methods, Figures, Data Availability Section)
In the figure legends: state number of times the experiment was **replicated** in laboratory.	Yes	Figure legends
In the figure legends: define whether data describe **technical or biological replicates**.	Yes	Figure legends

Ethics

Ethics	Information included in the manuscript?	In which section is the information available? (Reagents and Tools Table, Materials and Methods, Figures, Data Availability Section)
Studies involving **human participants**: State details of **authority granting ethics approval** (IRB or equivalent committee(s), provide reference number for approval.	Not Applicable	
Studies involving **human participants**: Include a statement confirming that **informed consent** was obtained from all subjects and that the experiments conformed to the principles set out in the WMA Declaration of Helsinki and the Department of Health and Human Services Belmont Report.	Not Applicable	
Studies involving **human participants:** For publication of **patient photos**, include a statement confirming that consent to publish was obtained.	Not Applicable	
Studies involving experimental **animals**: State details of **authority granting ethics approval** (IRB or equivalent committee(s), provide reference number for approval. Include a statement of compliance with ethical regulations.	Yes	Materials and Methods
Studies involving **specimen and field samples:** State if relevant **permits** obtained, provide details of authority approving study; if none were required, explain why.	Not Applicable	

Dual Use Research of Concern (DURC)	Information included in the manuscript?	In which section is the information available? (Reagents and Tools Table, Materials and Methods, Figures, Data Availability Section)
Could your study fall under dual use research restrictions? Please check biosecurity documents and list of **select agents and toxins** (CDC): https://www.selectagents.gov/sat/list.htm	Not Applicable	
If you used a select agent, is the security level of the lab appropriate and reported in the manuscript?	Not Applicable	
If a study is subject to dual use research of concern regulations, is the name of the **authority granting approval and reference number** for the regulatory approval provided in the manuscript?	Not Applicable	

Reporting

The MDAR framework recommends adoption of discipline-specific guidelines, established and endorsed through community initiatives. Journals have their own policy about requiring specific guidelines and recommendations to complement MDAR.

Adherence to community standards	Information included in the manuscript?	In which section is the information available? (Reagents and Tools Table, Materials and Methods, Figures, Data Availability Section)
State if relevant guidelines or checklists (e.g., **ICMJE, MIBBI, ARRIVE, PRISMA**) have been followed or provided.	Yes	Materials and Methods
For **tumor marker prognostic studies**, we recommend that you follow the **REMARK** reporting guidelines (see link list at top right). See author guidelines, under 'Reporting Guidelines'. Please confirm you have followed these guidelines.	Not Applicable	
For **phase II and III randomized controlled trials**, please refer to the **CONSORT** flow diagram (see link list at top right) and submit the CONSORT checklist (see link list at top right) with your submission. See author guidelines, under 'Reporting Guidelines'. Please confirm you have submitted this list.	Not Applicable	

Data Availability

Data availability	Information included in the manuscript?	In which section is the information available? (Reagents and Tools Table, Materials and Methods, Figures, Data Availability Section)
Have **primary datasets** been deposited according to the journal's guidelines (see 'Data Deposition' section) and the respective accession numbers provided in the Data Availability Section?	Not Applicable	
Were **human clinical and genomic datasets** deposited in a public access-controlled repository in accordance to ethical obligations to the patients and to the applicable consent agreement?	Not Applicable	
Are **computational models** that are central and integral to a study available without restrictions in a machine-readable form? Were the relevant accession numbers or links provided?	Not Applicable	
If publicly available data were reused, provide the respective **data citations in the reference list**.	Not Applicable	
